# Dropout Rate in Digital Health Interventions for the Prevention of Skin Cancer: Systematic Review, Meta-analysis, and Metaregression

**DOI:** 10.2196/42397

**Published:** 2022-12-09

**Authors:** Juan-Carlos Hernández-Rodríguez, Cristina García-Muñoz, Juan Ortiz-Álvarez, Francesc Saigí-Rubió, Julián Conejo-Mir, Jose-Juan Pereyra-Rodriguez

**Affiliations:** 1 Department of Dermatology Virgen del Rocío University Hospital Seville Spain; 2 Department of Nursing and Physical Therapy University of Cadiz Cadiz Spain; 3 Faculty of Health Sciences Universitat Oberta de Catalunya Barcelona Spain; 4 Department of Medicine University of Seville Seville Spain

**Keywords:** skin cancer, digital health, dropout, prevention, systematic review, meta-analysis, meta analyses, review methodology, cancer, skin, dermatology, attrition, digital intervention, digital treatment, eHealth, randomized controlled trial, RCT

## Abstract

**Background:**

Digital strategies are innovative approaches to the prevention of skin cancer, but the attrition following this kind of intervention needs to be analyzed.

**Objective:**

The aim of this paper is to assess the dropouts from studies focused on digital strategies for the prevention of skin cancer.

**Methods:**

We conducted this systematic review with meta-analyses and metaregression according to the PRISMA (Preferred Reporting Items for Systematic Reviews and Meta-Analyses) statements. Search terms for skin cancer, digital strategies, and prevention were combined to search PubMed, Scopus, Web of Science, CINAHL, and Cochrane Library from inception until July 2022. Randomized clinical trials that reported dropouts of participants and compared digital strategies with other interventions to prevent skin cancer in healthy or disease-free participants were included. Two independent reviewers extracted data for analysis. The Revised Cochrane Collaboration Bias tool was employed. We calculated the pooled dropout rate of participants through a meta-analysis of proportions and examined whether dropout was more or less frequent in digital interventions against comparators via an odds ratio (OR) meta-analysis. Data were pooled using a random-effects model. Subgroup meta-analyses were conducted in a meta-analysis of proportions and OR meta-analysis to assess the dropout events when data were sorted by digital interventions or control comparator. A univariate metaregression based on a random-effects model assessed possible moderators of dropout. Participants’ dropout rates as pooled proportions were calculated for all groups combined, and the digital and comparator groups separately. OR>1 indicated higher dropouts for digital-based interventions. Metaregressions were performed for age, sex, length of intervention, and sample size.

**Results:**

A total of 17 studies were included. The overall pooled dropout rate was 9.5% (95% CI 5.0-17.5). The subgroup meta-analysis of proportions revealed a dropout rate of 11.6% for digital strategies (95% CI 6.8-19.0) and 10.0% for comparators (95% CI 5.5-17.7). A trend of higher dropout rates for digital strategies was observed in the overall (OR 1.16, 95% CI 0.98-1.36) and subgroup OR meta-analysis, but no significant differences were found between the groups. None of the covariates moderated the effect size in the univariate metaregression.

**Conclusions:**

Digital strategies had a higher dropout rate compared to other prevention interventions, but the difference was not significant. Standardization is needed regarding reporting the number of and reasons for dropouts.

**Trial Registration:**

International Prospective Register of Systematic Reviews (PROSPERO) CRD42022329669; https://www.crd.york.ac.uk/prospero/display_record.php?RecordID=329669

## Introduction

Digital strategies have experienced a boom in use in prevention programs for skin cancer in recent years. Primary and secondary prevention programs are the mainstay to reduce the incidence rate of skin cancer [1]. In fact, recent publications have stated a stabilization in melanoma incidence in young cohorts, due to governmental efforts to promote prevention programs [2]. Nonetheless, cases of melanoma will continue to rise in the coming years, primarily in older adults [3]. The continuous rise in the incidence of skin cancer in recent decades suggests a current global public threat [4,5].

Digital strategies seem to be more effective in the prevention of skin cancer than other conventional strategies [6]. The former can be defined as interventions provided through a digital environment such as web-based interventions, smartphone apps, SMS text messaging, web-based videos, or wearable devices [7]. Digital approaches to the prevention of skin cancer present additional advantages such as feedback, interactivity, accessibility, and gamification, which make them suitable and attractive for stakeholders [8,9]. Conversely, possible drawbacks of digital strategies in dermatology could be their availability, financial aspects, reliability, security, confidentially, and lack of education and training of the user [10]. Given all these issues, the feasibility of randomized clinical trials (RCTs) in digital health research continues to be discussed [11,12]. However, digital strategies such as telemedicine in different areas of health care are expected to continue growing in the coming years [13].

The engagement of the patients with the prevention and digital strategies determines their effectiveness. Despite the increasing interest of researchers in implementing RCTs that analyze digital strategies, there is still no consensus in the literature on whether they positively or negatively influence the dropout and adherence of participants [14,15]. However, some authors have reported that the dropout rate was higher in digital strategies than analogue interventions [16,17]. Some of the reasons for the higher loss of participants could be the participant’s reluctance to join remote research studies and mistrust in sharing data [18].

Dropout or attrition is a constant challenge for researchers in RCTs and other longitudinal studies [19,20]. In addition, characteristics of the target population could influence attrition, because maintaining prevention behaviors in healthy participants could be challenging [21]. The absence of perceiving disease, geographical location, or accessibility are some of the factors that could lead to the failure of long-term prevention strategies [22,23]. Disentangling the factors and trend in dropouts in RCTs would help researchers develop future digital interventions for the prevention of skin cancer.

No previous studies have analyzed dropout in digital strategies for skin cancer prevention; therefore, our aim was to systematically assess and meta-analyze the existing RCTs to calculate the overall pooled dropout rate and to examine possible factors that could influence the dropout of users.

## Methods

### Protocol and Registration

We conducted this systematic review following the Preferred Reporting Items for Systematic Reviews and Meta-Analyses (PRISMA) guideline, 2020 [24]. Before the start of the study, the review protocol was registered in the International Prospective Register of Systematic Reviews (PROSPERO; CRD42022329669).

### Data Sources and Search Strategy

Two researchers (J-CH-R and CG-M) performed an independent electronic search in PubMed, Scopus, Web of Science, Cochrane Library, and CINAHL. The search included all records from the inception of the databases up to July 10, 2022. Search terms for digital strategies (*“virtual*,*” “online*,*” “web-based*,*” “internet-based*,*” “digital*,*” “e-Health*,*” “m-Health*,*” “App*,*”* and *“mApp”*), skin cancer (*“melanoma*,*” “cutaneous melanoma*,*” “malignant melanoma*,*”* and *“skin cancer”*), prevention (*“prevention”* and *“sun protection”*), and risk factors (*“tanned*,*” “sunburn*,*”* and *“UV exposure”*) were employed. These were combined using the Boolean operators “AND” and “OR.” Details of the search strategy can be found in Appendix S1 in Multimedia Appendix 1.

### Eligibility Criteria and Outcomes of Interest

We developed the eligibility criteria following the PICOS model (ie, patient, intervention, comparison, outcome, and study design) shown in Table 1.

**Table 1:** Eligibility criteria based on the PICOS^a^ model.

PICOS model	Inclusion criteria	Exclusion criteria
Population	Participants free of skin cancer during the study period	Participants with skin diseases during the study period
Intervention	Digital prevention strategies	Preventions approaches not focused on digital strategies
Comparator	Any type of comparator	Digital prevention strategies as comparator
Outcomes	Number of participants who dropout during the study period	Studies in which the dropout number was not reported, or indirect calculation was not allowed
Study design	Randomized controlled trials written in English	Any other type of study design

^a^PICOS: patient, intervention, comparison, outcome, and study design.

### Data Management and Selection Process

To manage data, Mendeley Desktop (version 1.19.8; Elsevier) was used to detect duplicates and carry out the screening process. Two independent researchers (J-CH-R and CG-M) screened records by title and abstract, and later performed a complete read of the studies to select those that met the mentioned criteria. Any disagreement was deliberated with a third researcher, J-JP-R.

### Assessment of Methodological Quality

We assessed methodological quality and risk of bias using The Cochrane Risk of Bias tool version 2 (ROB-2) [25]. This tool is composed of the following five domains: bias from randomization process, intended intervention, missing outcome data, measurement of outcomes, and selection of the reported results. The overall judgment is classified as “low,” “some concerns,” or “high” risk of bias. We also conducted subgroup analysis to determine how dropout events could be affected by the level of methodological quality and methodological threats such as blinding.

### Data Extraction and Qualitative Synthesis

The following data were extracted from the RCTs included in the systematic review: authors or year and country, study population, recruited sample, analyzed sample, sex, experimental and control intervention, dropout rate, reasons for dropouts, and length of intervention. When the number or rate of dropouts was not directly provided in the manuscripts, both were calculated.

### Quantitative Assessment of Data

A dropout was considered when a participant did not complete the intervention or follow-up period, after the randomization process. For studies that included more than 2 groups of intervention, we separately analyzed the comparison groups two by two. Dropout data were extracted from the text of the randomized controlled trials provided in either a flowchart, in the description of participants, in the results sections, or in the discussion.

To analyze data, we used the free software R Studio version 4.1.1. (R foundation for Statistical Computing) metafor (version 3.0-2) [26], *meta* (version 5.1-1) [27], and *dmetar* (version 0.0.9000) [28] packages. The analysis consisted of overall and subgroups proportion and odds ratio (OR)–based meta-analyses and metaregression.

A random-effects model was employed in all meta-analyses considering possible heterogeneity between our selected RCTs. Furthermore, heterogeneity was assessed with I^2^, with values exceeding 50% indicating large heterogeneity. The subgroup meta-analysis and metaregression was run when at least 3 arms of study were available.

The meta-analysis of proportions allowed us to calculate the overall pooled dropout rate with its 95% CI of all arms of the studies included in our review [29,30]. Additionally, a subgroup analysis was performed to calculate the pooled dropout rate for digital or comparator interventions and to determine which type of intervention resulted in the highest dropout rate. This analysis was complemented by an OR subgroup analysis ordered by digital or intervention comparator to determine whether the probability of losing the participants was greater in one group or another.

The OR meta-analysis evaluated whether the event (dropout) was more or less frequent in the digital or comparator intervention. When the OR was less than 1, dropouts were less likely in digital strategies. To assess the measure of effect on binary outcomes, the OR with a 95% CI was calculated, and the inverse variance method was used to adjust the pooled estimations to sparse data. The restricted maximum-likelihood estimator for τ^2^ estimated the variance among RCTs [31]. When studies reported zero events in one or all groups of intervention, we added a 0.5 continuity correction to the meta-analyses so that these studies could contribute to the overall sample size of the review [32]. The OR meta-analyses were conducted and subsequently described in terms of absolute values. The results of the meta-analyses were displayed in forest plots.

A sensitivity analysis was carried out to detect how studies influenced the effect size. When a study was identified as an outlier based on the dropout variable, it was removed from the analysis. Furthermore, to confirm previous results, we performed an exploratory analysis using the L’Abbé, Baujat plot, Leave-One-Out meta-analysis, and influence plot.

A univariate metaregression analysis based on a random-effects model assessed the continuous variables of age, female percentage, male percentage, length of intervention in months, and sample size as covariates of the occurrence of dropouts. These predictors were selected to determine how the characteristics of the participants and interventions could influence dropouts [33]. Bubble plots were used to illustrate how a covariate modified the effect size in the metaregression analysis.

### Publication Bias Assessment

We examined the effects of small studies and publication bias based on the symmetry of the contour-enhanced funnel plot. The Harbord and Egger bias test were used to confirm the absence of asymmetry in the funnel plot (*P*>.05).

## Results

### Study Selection and Methodological Quality Assessment

**Figure 1:**
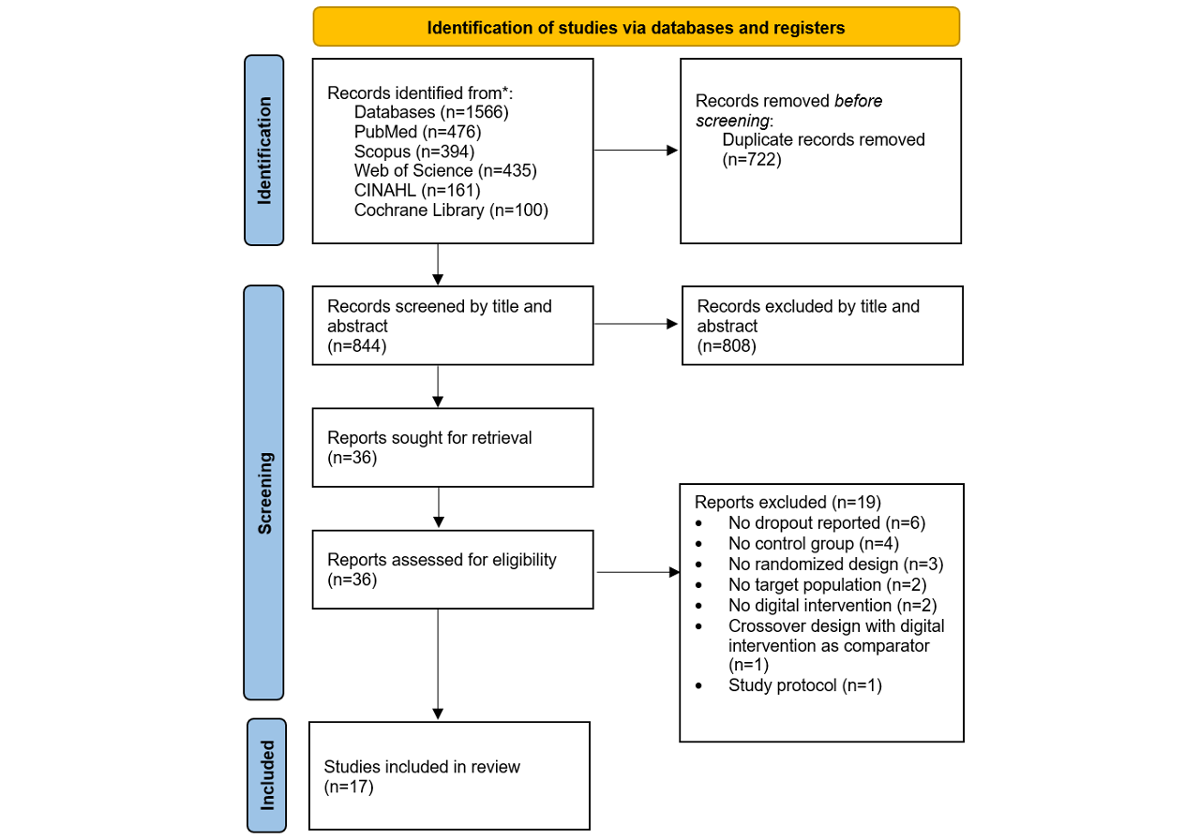
Flow diagram of trials selection based on PRISMA (Preferred Reporting Items for Systematic Reviews and Meta-Analyses) 2020 guidelines.

A total of 1566 studies were identified in the database search. After removing duplicates, the screening process, and complete reading of the records that met the eligibility criteria, 17 RCTs were finally included in the review [34-50]. The complete process is shown in Figure 1. Details of the excluded records are presented in Table S1 in Multimedia Appendix 1.

Regarding methodological quality, 14 (82%) of 17 RCTs showed “some concerns” based on the summary score of ROB-2. Moreover, 2 (12%) RCTs [44,47] showed a “low“ risk of bias, and only 1 (6%) had a “high” risk of bias [49] (Figure S1 in Multimedia Appendix 1). The latter showed a “high” risk of bias because baseline differences between groups were observed.

Regarding the subgroup analyses, an analysis sorted by participants’ blinding condition could not be performed because most of the studies were not blind or the blinding was not clearly specified. The subgroup meta-analysis sorted by the ROB-2 scores (Figure S2 in Multimedia Appendix 1) showed that a “low” overall score could indicate lower attrition in nondigital prevention strategies. However, due to the limited number of “low”-risk studies, the results should be interpreted with caution.

### Study Design and Population Characteristics

A sample of 6593 healthy participants and people free of disease during the study period was analyzed. The age of the participants ranged from 12.6 to 54.3 years. The digital strategies used in the included RCTs were web-based interventions in 8 studies [35,40,42,43,46,48-50], 6 involved apps [36-38,44,45,47], 3 involved SMS [35,36,39], 2 involved video [34,41], and 1 involved a wearable device [47]. Conversely, the comparator groups involved no intervention in 11 studies [35,37,43-48,50] and active controls in 6 studies [34,36,38,39,41,49].

The total number of dropouts for all arms of the included studies was 1120, with 681 (60.80%) in experimental interventions and 439 (39.20%) in controls. The reason for the dropout of participants was reported as loss during follow-up in 9 of the 17 RCTs [34,37,38,40-43,47,49] and not answering the final questionnaire in 4 studies [39,44,45,50]; 2 studies did not report the reason for dropout [35,48]. The main characteristics of the studies are displayed in Table 2.

**Table 2:** Summary of the included studies in the systematic review.

Source	Population	Recruited or analyzed (n)	Percentage of sex, age (years), or mean (SD)	Experimental intervention	Comparator intervention	Dropout rate (%)	Reason for dropouts (EG/CG^a^)	Length of intervention (months)
Armstrong et al, 2011 [34]; United States	English speakers aged >18 years	EG: 47/43; CG: 47/40; n=94	Female: 50%; male: 50%; 37.2 years	Online video addressing how sunscreen works to protect skin	Active (brochure)	EG: 8.5% (4/47); CG: 14.9% (7/47)	Lost to follow-up	3
Böttcher et al, 2019 [35]; Germany	Young organ transplant recipients	EG1: 44/39; EG2: 49/40; CG: 44/33; n=137	Female: 44.5%; male: 55.5%; 12.6 years	EG1: SMS text message providing sun protection advice; EG2: WBI^b^ with sun protection training	No intervention (waitlist)	EG1: 11.4% (5/44); EG2: 18.4% (9/49); CG: 25.0% (11/44)	N/R^c^	12
Bowen et al, 2019 [36]; United States	First-degree relatives of melanoma cases	EG: 157/141; CG: 156/137; n=313	Female: 63.6%; male: 36.4%; 51.3 years	WBI with weekly messages of melanoma prevention behaviors	No intervention (waitlist)	EG: 10.2% (16/157); CG: 12.2% (19/156)	Lost to follow-up	12
Brinker et al, 2020 [37]; Brazil	Secondary school pupils	EG: 734/734; CG: 839/839; n=1573	Female: 51.6%; male: 48.4%; 15.9 (SD 1.3) years	App that modifies a selfie according to different levels of UV exposure for future 5 to 25 years based on individual skin type	No intervention	EG: 17.3% (127/734); CG: 6.20% (52/839)	Lost to follow-up	6
Buller et al, 2015 [38]; United States	Adults aged >18 years owning a smartphone	EG: 96/89; CG: 106/104; n=202	Female: 73.5%; male: 26.5%; 33.3 (SD 9.8) years	App giving feedback on sun protection and alerted users to apply or to reapply sunscreen and to get out of the sun	No intervention	EG: 7.3% (7/96); CG: 1.9% (2/106)	Lost to follow-up and survey not completed	3
Craciun et al, 2011 [39]; United Kingdom, Germany, Portugal, and Romania	Female volunteers	EG1: 74/74; EG2: 70/70; CG: 61/61; n=205	Male: 0%; female: 100%; 25.1 (SD 8.7) years	EG1: WBI volitional theory–based; EG2: WBI motivational theory–based	No intervention	0%	Not applied	1
Hacker et al, 2018 [40]; Australia	Young adults aged 18-35 years	EG1: 41/35; EG2: 42/36; CG: 41/36; n=124	Female: 65.8%; male: 31.5%; 25.8 years	EG1: app that displays the daily UV index and gives sun protection advice; EG2: wearable with UV dosimeter	No intervention	EG1: 14.6% (6/41); EG2: 14.3% (6/42); CG: 12.2% (5/41)	Lost to follow-up	3
Heckman et al, 2016 [41]; United States	Adults aged 18-25 years	EG1: 287/195; EG2: 338/205; CG: 340/229; n=965	Female: 66.1%; male: 33.9%; 21.8 (SD 2.2) years	EG1: WBI with a tailored intervention based on the Integrative Model of Behavioral Prediction; EG2: WBI with the Skin Cancer Foundation website	No intervention	EG1: 32.1% (92/287); EG2: 39.4% (133/338); CG: 32.7% (111/340)	N/R	3
Hillhouse at al, 2017 [42]; United States	Female adolescents	EG: 214/182; CG: 229/206; n=443	Female: 100%; male: 0%; 15.2 (SD 2.0) years	WBI to reduce IT^d^ motivations	Active (placebo)	EG: 15.9% (32/214); CG: 10.1% (23/229)	Lost to follow-up	6
Manne et al, 2021 [43]; United States	Participants at increased risk for melanoma aged 18-89 years	EG: 56/43; CG: 60/56; n=116	Female: 69.8%; male: 30.2%; 51.1 (SD 15.2) years	WBI to improve SSE^e^ and sun protection	No intervention	EG: 76.8% (13/56); CG: 93.3% (4/60)	Survey not completed	3
Marek et al, 2018 [44]; United States	Adults aged ≥18 years	EG1: 18/18; EG2: 17/17; EG3: 17/17; CG: 17/17; n=69	Female: 61.1%; male: 38.9%; 54.3 (SD 13.9) years	EG1: app allowing total body photography; EG2: SMS to remind SSE; EG3: SMS+ accountability partner	Active (accountability partner)	0%	Not applied	6
Reilly et al, 2021 [45]; Scotland	Adults aged >18 years who survived stage 0-2C primary cutaneous melanoma	EG: 121/82; CG: 119/86; n=240	N/A^f^	App to encourage and improve SSE	No intervention	EG: 32.2% (39/121); CG: 27.7% (33/119)	Lost to follow-up	12
Robinson et al, 2016 [46]; United States	Kidney transplant recipients	EG: 84/78; CG: 86/83; n=170	Female 40.6%; male: 59.4%; 50.0 years	App with educational sun protection content	Active (usual education)	EG: 7.1% (6/84); CG: 3.5% (3/86)	Lost to follow-up	1.5
Robinson et al, 2021 [47]; United States	Female adults	EG: 494/390; CG: 495/414; n=989	Female: 100%; male: 0%; 47.0 years	SMS to remind SSE	Active (brochure)	EG: 21.1% (104/494); CG: 16.4% (81/495)	Survey not completed and discontinued intervention (EG)	3
Stapleton et al, 2015 [48]; United States	Female adults aged 18-25 years with IT in the past 12 months	EG: 94/74; CG: 93/85; n=186	Female: 100%; male: 0%; 19.8 (SD1.4) years	WBI with psychoeducational content to reduce IT	No intervention	EG: 8.5% (8/94); CG: 8.6% (8/93)	No response	1.5
Tsai et al, 2017 [49]; United States	Adults aged ≥18 years	EG: 71/42; CG: 72/34; n=143	Female: 74.1%; male: 25.9%; 42.3 years	Online melanoma video tutorial + brochure	Active (brochure)	EG: 40.8% (29/71); CG: 52.8% (38/72)	Lost to follow-up	1
Vuong et al, 2018 [50]; Australia	General practice patients	EG: 134/89; CG: 138/96; n=272	Female: 71.7%; male: 28.3%; 45.5 years	WBI with tailored melanoma risk assessment and prevention + usual education	Active (usual education)	EG: 33.9% (45/134); CG: 30.4% (42/138)	Lost to follow-up	1.5

^a^CG: comparator group; EG: experimental group.

^b^WBI: web-based intervention.

^c^N/R: not reported.

^d^IT: indoor tanning.

^e^SSE: skin self-examination.

^f^N/A: not applicable.

### Sensitivity Analysis

The initial sensitivity analysis included a total of 23 arms from the randomized controlled trials of the review. After the sensitivity analysis, the study conducted by Brinker et al [44] was removed because it was identified as an outlier that influenced the effect size. The details of the sensitivity analysis are shown in Figures S3-S6 in Multimedia Appendix 1. Figure S7 in Multimedia Appendix 1 shows a funnel plot with absence of asymmetry, as confirmed by the Harbord test (*P*=.66) and Egger bias test (*P*=.69).

### Meta-analysis of Proportions

**Figure 2:**
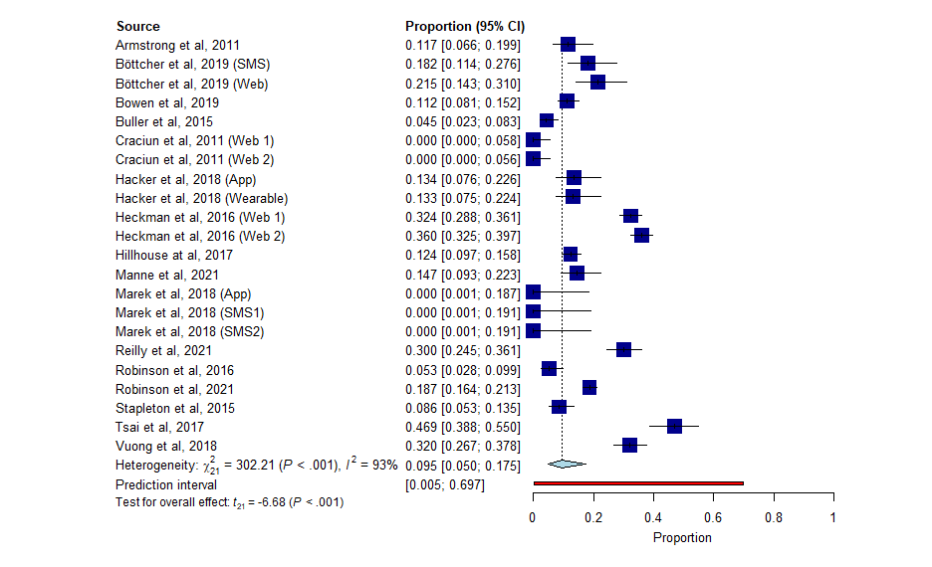
Forest plot of overall meta-analysis of proportions for all groups of studies.

The meta-analysis of proportions included 22 arms (*k*) of study and 2610 subjects among whom there were 419 dropouts. An overall pooled dropout rate of 9.5% (95% CI, 5.0-17.5) was calculated (Figure 2; [34-36,38-50]). In the subgroup meta-analysis, digital strategies showed a higher dropout rate of 11.6% (95% CI 6.8-19.0) compared to 10.0% (95% CI 5.5-17.7) in the comparators. These results are displayed in forest plots, respectively, in Figures S8 and S9 in Multimedia Appendix 1.

### Odds Ratio Meta-analysis

**Figure 3:**
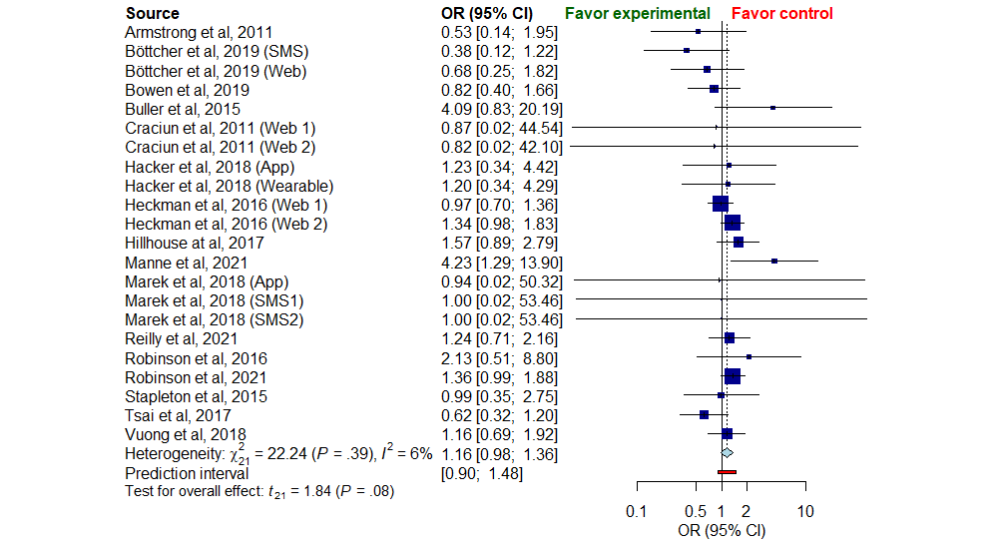
Forest plot of overall odds ratio meta-analysis for all groups of studies.

A slight trend for a higher number of dropouts was observed in digital strategies with an OR of 1.16 (95% CI 0.98-1.36), but there were no significant differences between the experimental and control approaches (*P*=.39). The *I*^2^ was 6% (95% CI 0-38) indicating a lack of heterogeneity between the studies analyzed for the overall and subgroup meta-analysis (Figure 3; [34-36,38-50]).

We performed a meta-analysis of subgroups divided by the type of digital strategy and the comparison groups. Only the strategies that were analyzed in more than two RCTs were included in the OR meta-analysis. As Figure S10 in Multimedia Appendix 1 shows, none of the digital interventions assessed differed significantly in the number of dropouts compared with the comparator strategies. The OR score was 0.88 for SMS (95% CI, 0.30-2.53), 1.17 for web-based interventions (95% CI 0.94-1.47), and 1.44 for Apps (95% CI 0.88-2.35). Our findings in the comparator subgroup analysis showed no significant differences, even when comparing digital strategies with active control (OR 1.13; 95% CI 0.82-1.54) or no-intervention groups (OR 1.14; 95% CI 0.90-1.44; Figure S11 in Multimedia Appendix 1).

### Metaregression

Univariate metaregression analysis (Table 3) for age, female percentage, male percentage, and length of intervention in months and sample size did not show any significant association with the effect size of the study. Metaregression bubble plots for these analyses are presented in Figures S12-S16 in Multimedia Appendix 1.

**Table 3:** Univariate metaregression analysis.

Covariate	Coefficient (95% CI)^a^	SE	*t* value	*P* value
Age	0.05 (–0.01 to 0.02)	0.24	–0.08	.53
Percentage of female	0.008 (–0.001 to 0.018)	0.004	1.78	.09
Percentage of male	–0.008 (–0.02 to 0.001)	0.005	–1.79	.09
Length of intervention (months)	–0.023 (–0.07 to 0.03)	0.023	–0.98	.34
Sample size	0.0004 (–0.0002 to 0.0009)	0.0002	1.45	.16

^a^According to the random-effects model.

## Discussion

### Principal Findings

This systematic review synthesizes information on the attrition of RCTs based on eHealth interventions for the prevention of skin cancer. Quantitative analysis evaluated the pooled dropout rate and dropout OR, in addition to moderators that could influence the dropout of subjects in the meta-analyzed RCTs. Although the digital strategies employed within studies used different platforms or devices, all of them were focused on skin cancer prevention and were supervised by expert dermatologists.

The meta-analysis of proportions showed a pooled dropout rate of 9.5%, with a dropout rate of 11.6% and 10.0% for the eHealth interventions and comparators, respectively. These results are in line with the findings by Walters et al [51], who reviewed the retention in RCTs of health technology programs in the United Kingdom. This review established that there was a dropout rate of up to 11% in a significant proportion of RCTs. Dropout rates of 5% are likely to introduce bias, while if 20% is exceeded, this could affect the validity of the study due to insufficiency during data analysis [52,53]. No background research was found performing similar analyses in the dermatology literature, so the comparison of rates was not viable.

Eysenbach et al [20] hypothesized that the nature of digital strategies tends to a higher loss of participants, a phenomenon called the “Law of attrition.” Although a slightly higher dropout rate was observed in digital strategies compared with comparator groups in our proportion and OR meta-analysis, the difference was not significant. Our findings refute the “Law of attrition” in those studies that aim to prevent skin cancer through these innovative interventions.

Previous systematic reviews, such as Bevens et al [54], focused on the analysis of attrition of digital strategies in people with multiple sclerosis and found no significant differences between dropout rates in participants allocated to digital or control interventions. Although our findings are in line with these previous findings, the target population and research conditions differed from ours, so comparison of findings are difficult.

As in the overall OR meta-analysis, the subgroup meta-analysis sorted by type of digital strategy and comparators found no significant differences in dropout rate. Only SMS text messaging presented a lower odd of dropout compared with other digital interventions, but without statistical significance. Reminder-based interventions such as SMS seem to promote adherence in chronic conditions, but further research is still needed [55]. It is noteworthy that the dropout rates in participants allocated to no intervention showed losses similar to digital strategies, reflecting the prior expectation that they could be affected by nonexperimental factors and the loss of perspective of therapeutics goals [56].

Our metaregression found that none of the covariates moderated the interventions’ effect size. Nonetheless, Torous et al [17] obtained higher dropout rates in studies with larger sample sizes that used apps for depressive symptoms, possibly related to a lower rate of individual follow-up and feedback from subjects.

In addition to the moderator analysis, assessment of the reasons for dropping out could be a way to identify barriers to reduce attrition in future RCTs. However, the lack of transparency and homogeneity in reporting reasons for participants’ dropout in the studies included in this review made the aforementioned task challenging. The main reported cause of attrition in our RCTs was loss to follow-up, but this aspect did not show the real reason for the loss of participants.

### Research Implications

As previously mentioned, dropout could threaten internal or external validity in studies. We recommend that researchers use our overall pooled dropout rate to calculate the sample size of future trials, avoiding possible threats. The overrecruitment of 10.1% in the sample size of RCTs may be a suitable way to overcome external validity risks [57,58].

Although our OR meta-analysis showed no differences in attrition between digital strategies and comparator interventions, in order to obtain conclusive results that can be turned into daily clinical practice, we point out the need for further research with head-to-head comparison between digital and conventional interventions (eg, education programs or brochures) for the prevention of skin cancer [59]. Dropout rates have previously been directly related to the acceptability and feasibility of the intervention [60,61].

Given the scarce information and lack of transparency provided by studies when reporting the number of and reasons for dropouts, a deep change in the research framework is needed. To overcome this obstacle, relevant guidelines such as Consolidated Standards of Reporting Trials report the need to detail the reasons and the number of participants lost during the study period [62,63]. Accurately following these guidelines would pave the way for researchers to find suitable dropout prevention plans. Previous literature, based on user experience with digital strategies, indicates that reliability, lack of technological education, lack of satisfaction with intervention, and sparse human feedback seem to be the main barriers to their use [63-65]. We encourage future researchers who aim to develop a digital strategy or perform RCT protocols to implement solutions to the mentioned barriers such as gamification, tailored and customizable e-interventions, personalized feedback, or programmed reminders (eg, mail and SMS). The gamification principles of meaningful purpose, meaningful choice, supporting player archetypes, feedback, and visibility proposed by Floryan et al [66] could enhance the user experience and engagement within digital health interventions. Gamification could increase motivation, reinforce learning objectives, and increase enjoyment and positive experiences in dermatological education and prevention approaches [9]. Likewise, programmed reminders are an effective way to promote prevention habits, highlighted by the use of text messages in dermatology [64]. Reminders associated with professional supervision have shown even greater results in prevention programs [67].

Given that RCTs are the first step required to translate research results into clinical settings, success in decreasing the number of participants dropping out within the research context could improve long-term engagement in digital programs for the prevention of skin cancer.

### Strengths and Limitations

This review has several strengths. Our study provided an initial analysis of the dropout from RCTs to prevent skin cancer through digital strategies. Our computed rates could help calculate sample sizes in future studies. We performed a sensitivity analysis that helped us detect outliers and confirm the absence of publication bias. Moreover, the subgroups and metaregression analyses allowed us to understand how loss of participants could be modified by different predictors.

The main limitation of our review is that potential literature from other databases with non-English records could have been missing. Furthermore, our outcomes may have been conditioned by the heterogeneity of the experimental interventions in the included studies. Some of the studies compared digital strategies with no intervention, so we cannot assert that dropouts from these groups could be related to external factors. Evidence from the subgroup meta-analysis sorted by an active comparator group should be interpreted with caution because of the low number of analyzed studies; further research is needed to obtain strong evidence. We were unable to propose tailored advice to improve retention for this kind of RCT owing to the sparse information on reasons for dropout provided by the authors.

### Conclusions

This systematic review and meta-analysis calculated an overall pooled participant dropout rate of 9.5% (95% CI 5.0-17.5), which should be considered in the calculation of sample size in RCTs aimed at preventing skin cancer using digital health interventions. Although a slightly higher pooled dropout rate was recorded for digital strategies, the OR-based meta-analysis did not show significant differences against the comparator groups. Our meta-analyses of subgroups sorted by digital and comparator interventions did not present significant statistical differences. Age, sex, length of the intervention, and sample size did not modify the effect size, so they were not moderators of dropout. We highlight the need to follow the guidelines and standardize reporting of the number of and reasons for participants’ dropout because this will be the only effective way to design a successful plan to reduce the loss of participants in studies that analyze digital approaches to prevent skin cancer.

## Appendix

Multimedia Appendix 1 Supplemental material.

## Abbreviations

OR: odds ratio
PRISMA: Preferred Reporting Items for Systematic Reviews and Meta-Analyses
PROSPERO: International Prospective Register of Systematic Reviews
RB-2: The Cochrane Risk of Bias tool version 2
RCT: randomized clinical trial
