# Supplementary material for: Dropout Rate in Digital Health Interventions for the Prevention of Skin Cancer: Systematic Review, Meta-analysis, and Metaregression
Source: J Med Internet Res. 2022 Dec 9;24(12):e42397. doi: 10.2196/42397 (PMC9789500; doi:10.2196/42397)
Supplement: Multimedia Appendix 1 [file jmir_v24i12e42397_app1.docx]

**Multimedia Appendix 1**

Appendix S1. Search strategy

Table S1. Excluded studies and reasons

Figure S1. Cochrane Risk of Bias Tool version 2

Figure S2. Subgroup meta-analysis sort by the scored of ROB-2 tool

Figure S3. L’Abbé plot

Figure S4. Baujat plot

Figure S5. Leave-One-Out meta-analysis

Figure S6. Influence graph

Figure S7. Contour enhanced-funnel plot by log odds ratio

Figure S8. Forest plot of proportion subgroup meta-analysis digital health interventions groups

Figure S9. Forest plot of proportion subgroup meta-analysis comparator groups

Figure S10. Forest plot of odds ratio-based subgroup meta-analysis digital health interventions groups

Figure S11. Forest plot of odd ratio-based subgroup meta-analysis comparator groups

Figure S12. Bubble plot of association of Participants’ dropout and age

Figure S13. Bubble plot of association of Participants’ dropout and percentage of females

Figure S14. Bubble plot of association of Participants’ dropout and percentage of males

Figure S15. Bubble plot of association of Participants’ dropout and length of intervention (months)

Figure S16. Bubble plot of association of Participants’ dropout and sample size

**Appendix S1.** Search strategy

| **Nº** | **Search term** |
| --- | --- |
| #1 | virtual |
| #2 | online |
| #3 | web-based |
| #4 | internet-based |
| #5 | digital |
| #6 | e-Health |
| #7 | m-Health |
| #8 | App |
| #9 | mApp |
| #10 | melanoma |
| #11 | cutaneous melanoma |
| #12 | malignant melanoma |
| #13 | skin cancer |
| #14 | prevention |
| #15 | sun protection |
| #16 | tanned |
| #17 | sunburn |
| #18 | UV exposure |

| **PubMed** | **Results** |
| --- | --- |
| (#1 OR #2 OR “#3” “#4” OR #5 OR “#6” OR “#7” OR “#8” OR “#9”) AND (#10 OR “#11” OR “#12” OR “#13”) AND (#14 OR “#15” OR #16 OR #17 OR “#18”) | 476 |
| **Scopus** | **Results** |
| (#1 OR #2 OR “#3” “#4” OR #5 OR “#6” OR “#7” OR “#8” OR “#9”) AND (#10 OR “#11” OR “#12” OR “#13”) AND (#14 OR “#15” OR #16 OR #17 OR “#18”) | 394 |
| **Web of Science** | **Results** |
| (#1 OR #2 OR “#3” “#4” OR #5 OR “#6” OR “#7” OR “#8” OR “#9”) AND (#10 OR “#11” OR “#12” OR “#13”) AND (#14 OR “#15” OR #16 OR #17 OR “#18”) | 435 |
| **CINHAL** | **Results** |
| (#1 OR #2 OR “#3” “#4” OR #5 OR “#6” OR “#7” OR “#8” OR “#9”) AND (#10 OR “#11” OR “#12” OR “#13”) AND (#14 OR “#15” OR #16 OR #17 OR “#18”) | 161 |
| **COCHRANE LIBRARY:** | **Results** |
| (#1 OR #2 OR “#3” “#4” OR #5 OR “#6” OR “#7” OR “#8” OR “#9”) AND (#10 OR “#11” OR “#12” OR “#13”) AND (#14 OR “#15” OR #16 OR #17 OR “#18”) | 100 |

**Table S1.** Excluded studies and reasons

| **No dropout reported (n =6)** |
| --- |
| 1. Agha-Mir-Salim L, Bhattacharyya A, Hart D, Lewandowska M, Spyropoulou E, Stinson L, et al. A randomised controlled trial evaluating the effectiveness of Facebook compared to leaflets in raising awareness of melanoma and harmful sun-related behaviour among young adults. Eur J cancer Prev. 2020; 29(1):89‐91. 2. Bleakley A, Jordan AB, Strasser AA, Lazovich D, Glanz K. Testing General Versus Specific Behavioral Focus in Messaging for the Promotion of Sun Protection Behaviors. Ann Behav Med. 2019; 54(2):108–18. 3. Bowen DJ, Burke W, Hay JL, Meischke H, Harris JN. Effects of web-based intervention on risk reduction behaviors in melanoma survivors. J Cancer Surviv. 2015; 9(2):279–86. 4. Chao LW, Enokihara MY, Silveira PS, Gomes SR, Böhm GM. Telemedicine model for training non-medical persons in the early recognition of melanoma. J Telemed Telecare. 2003; 9 Suppl 1: S4‐7. 5. Cho H, Song C (Chuck), Adams D. Efficacy and Mediators of a Web-Based Media Literacy Intervention for Indoor Tanning Prevention. J Health Commun. 2020; 25(2):105–14. 6. Manne S, Heckman CJ, Kashy D, Ritterband L, Thorndike F, Lozada C, et al. Moderators of the Effects of mySmartSkin, a Web-Based Intervention to Promote Skin Self-examination and Sun Protection Among Individuals Diagnosed With Melanoma. Ann Behav Med. 2022. |
| **No control group (n = 3):** |
| 1. Hughes-Barton D, Hutchinson A, Prichard I, Wilson C. Acceptability of online sun exposure awareness-raising interventions among young Australian women: an exploratory mixed-methods study. Health Promot Int. 2021; 36(2):374–83. 2. Jordan AB, Bleakley A, Alber JM, Lazovich D, Glanz K. Developing and Testing Message Strategies to Reduce Indoor Tanning. Am J Health Behav. 2020; 44(3):292–301. 3. Manne S, Buller D, Devine K, Heckman C, Pagoto S, Frederick S, et al. Sun Safe Partners Online: Pilot Randomized Controlled Clinical Trial. J Med Internet Res. 2020; 22(9): e18037. 4. Nittas V, Mütsch M, Frey T, Braun J, Puhan MA. Effectiveness of a tailored web app on sun protection intentions and its implications for skin cancer prevention: A randomized controlled trial. Oakley-Girvan I, editor. PLOS Digit Heal. 2022;1(5): e0000032. |
| **No randomized design (n =4):** |
| 1. Brinker TJ, Brieske CM, Schaefer CM, Petri MP, Sondermann W, Schadendorf D, et al. Photoaging Mobile Apps in School-Based Melanoma Prevention: Pilot Study. J Med Internet Res. 2017; 19(9):14. 2. Brinker TJ, Heckl M, Gatzka M, Heppt M V, Rodrigues HR, Schneider S, et al. A skin cancer prevention facial-aging mobile app for secondary schools in Brazil: Appearance-focused interventional study. JMIR mHealth uHealth. 2018; 6(3). 3. Tuong W, Armstrong AW. Effect of appearance-based education compared with health-based education on sunscreen use and knowledge: A randomized controlled trial. J Am Acad Dermatol. 2014; 70(4):665–9. 4. Eshtiaghi P, Khosravi-Hafshejani T, Sara G, Lui H, Kalia S. Assessment of sun-safety education behavior via spectrophotometric evaluation: A preliminary study. Photodermatol Photoimmunol Photomed. 2021. |
| **No target population (n=2):** |
| 1. Robinson JK, Jain N, Marghoob AA, McGaghie W, MacLean M, Gerami P, et al. A Randomized Trial on the Efficacy of Mastery Learning for Primary Care Provider Melanoma Opportunistic Screening Skills and Practice. J Gen Intern Med. 2018; 33(6):855–62. 2. Reilly F, Contstable L, Brant W, Rahman K, Durrani A, Burrows N, et al. Achieving integrated self-directed Cancer aftercare (ASICA) for melanoma: how a digital intervention to support total skin self-examination was used by people treated for cutaneous melanoma. BMC Cancer. 2021; 21(1):1217. |
| **No digital intervention (n=2):** |
| 1. Buller DB, Reynolds KD, Buller MK, Massie K, Berteletti J, Ashley J, et al. Parent reports of sun safety communication and behaviour for students in a randomised trial on a school policy implementation intervention. Aust N Z J Public Health. 2020; 44(3):208–14. 2. Buller DB, Buller MK, Meenan R, Cutter GR, Berteletti J, Eye R, et al. Design and baseline data of a randomized trial comparing two methods for scaling-up an occupational sun protection intervention. Contemp Clin Trials. 2020; 97: 106147. |
| **Crossover design with digital intervention as comparator (n=1):** |
| 1. Horsham C, Baade P, Kou K, O’Hara M, Sinclair C, Loescher LJ, et al. Optimizing Texting Interventions for Melanoma Prevention and Early Detection: A Latin Square Crossover RCT. Am J Prev Med. 2021; 61(3):348–56. |
| **Study protocol (n=1):** |
| 1. Coups EJ, Manne SL, Strickland PO, Hilgart M, Goydos JS, Heckman CJ, et al. Randomized controlled trial of the mySmartSkin web-based intervention to promote skin self-examination and sun protection behaviors among individuals diagnosed with melanoma: study design and baseline characteristics. Contemp Clin Trials. 2019; 83:117–27. |

**Figure S1.** Cochrane Risk of Bias Tool version 2


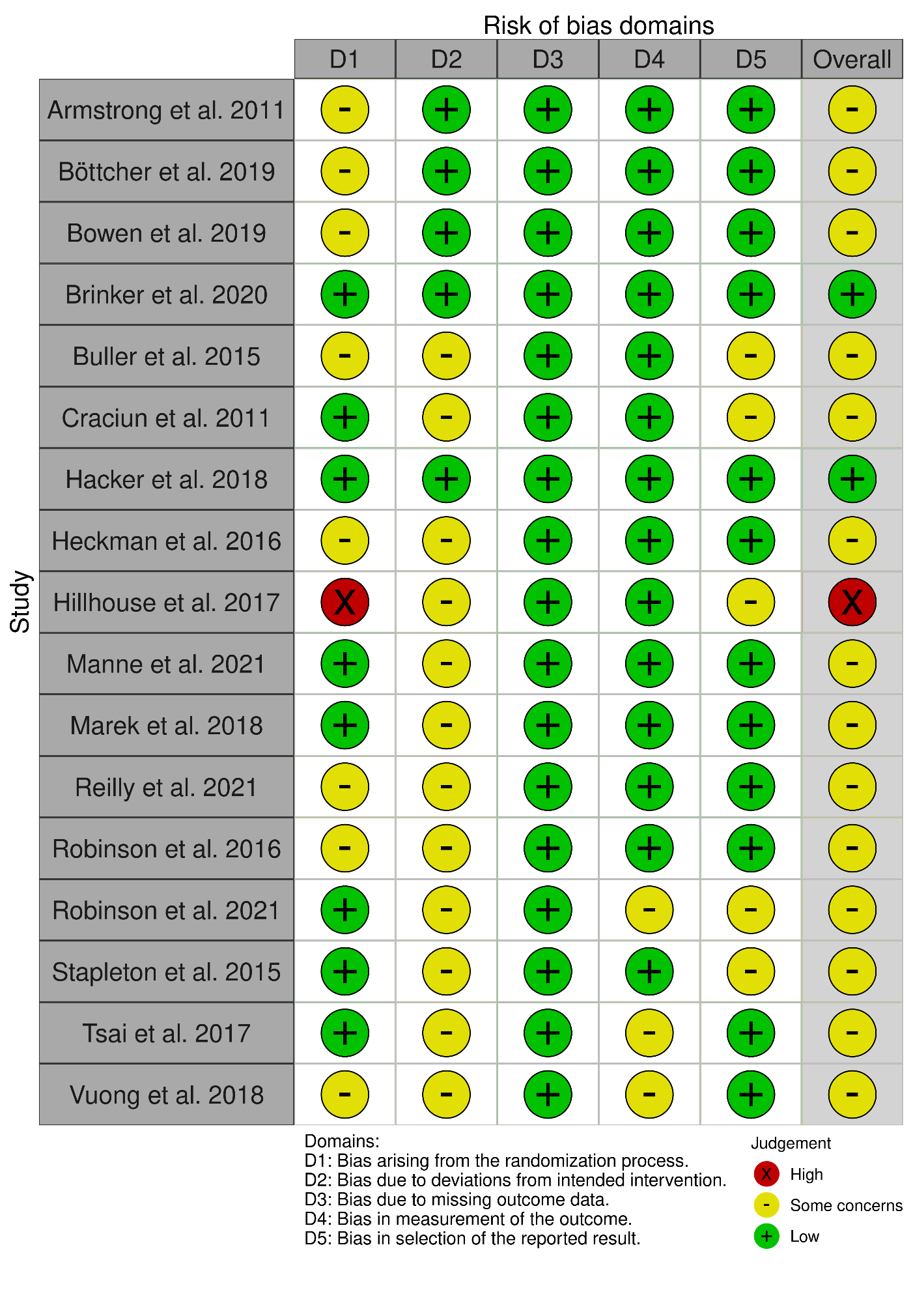


**Figure S2.** Subgroup meta-analysis sort by the scored of ROB-2 tool


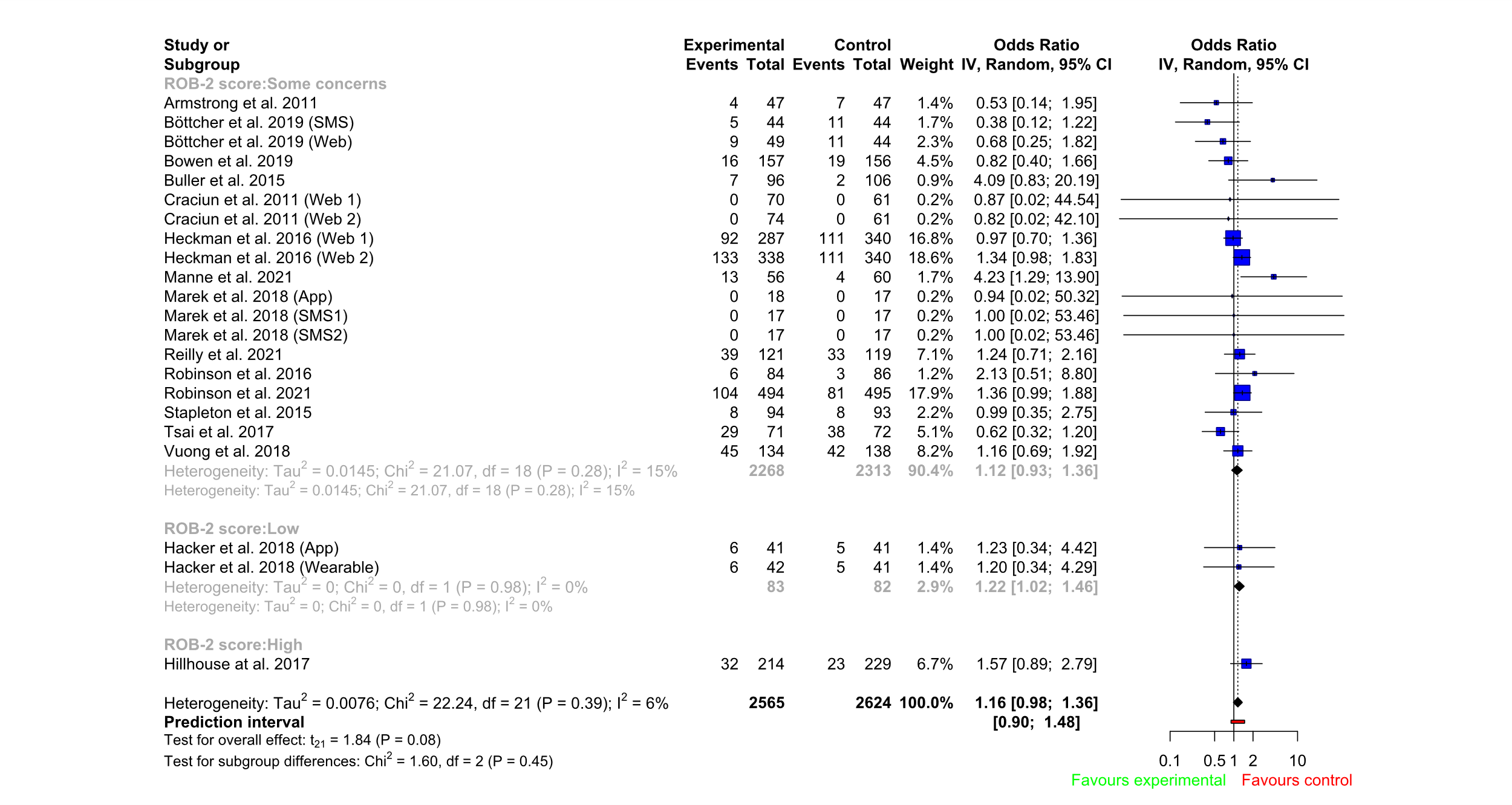


**Figure S3.** L’Abbé plot


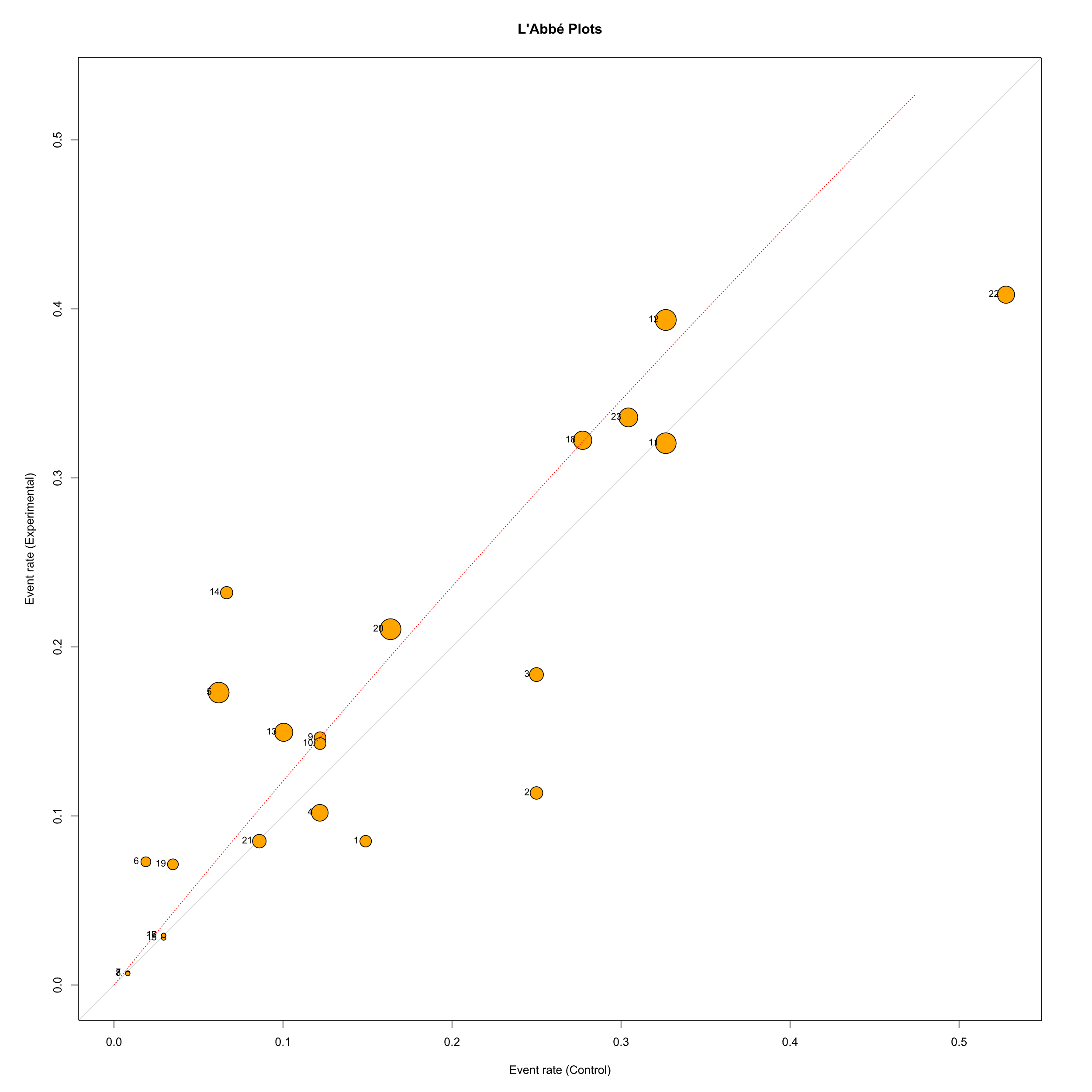


Studies ID: 1. Armstrong et al. 2011; 2. Böttcher et al. 2019 (SMS); 3. Böttcher et al. 2019 (Web); 4. Bowen et al. 2019; 5. Brinker et al. 2020; 6. Buller et al. 2015; 7. Craciun et al. 2011 (Web 1); 8. Craciun et al. 2011 (Web 2); 9. Hacker et al. 2018 (App); 10. Hacker et al. 2018 (Wearable); 11. Heckman et al. 2016 (Web 1); 12. Heckman et al. 2016 (Web 2); 13. Hillhouse at al. 2017; 14. Manne et al. 2021; 15. Marek et al. 2018 (App); 16. Marek et al. 2018 (SMS1); 17. Marek et al. 2018 (SMS2); 18. Reilly et al. 2021; 19. Robinson et al. 2016; 20. Robinson et al. 2021; 21. Stapleton et al. 2015; 22.Tsai et al. 2017; 23. Vuong et al. 2018

**Figure S4.** Baujat plot


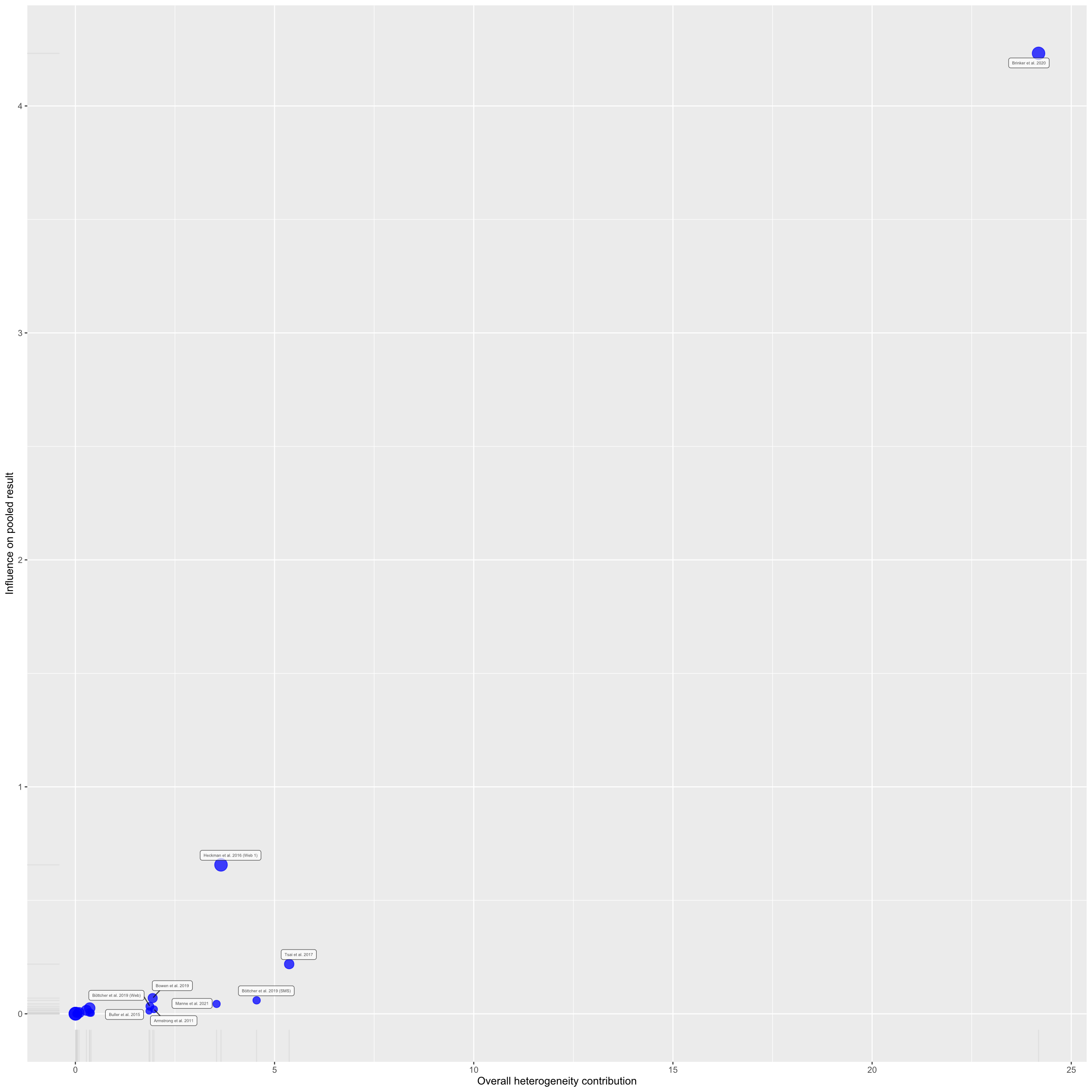


**Figure S5.** Leave-One-Out meta-analysis


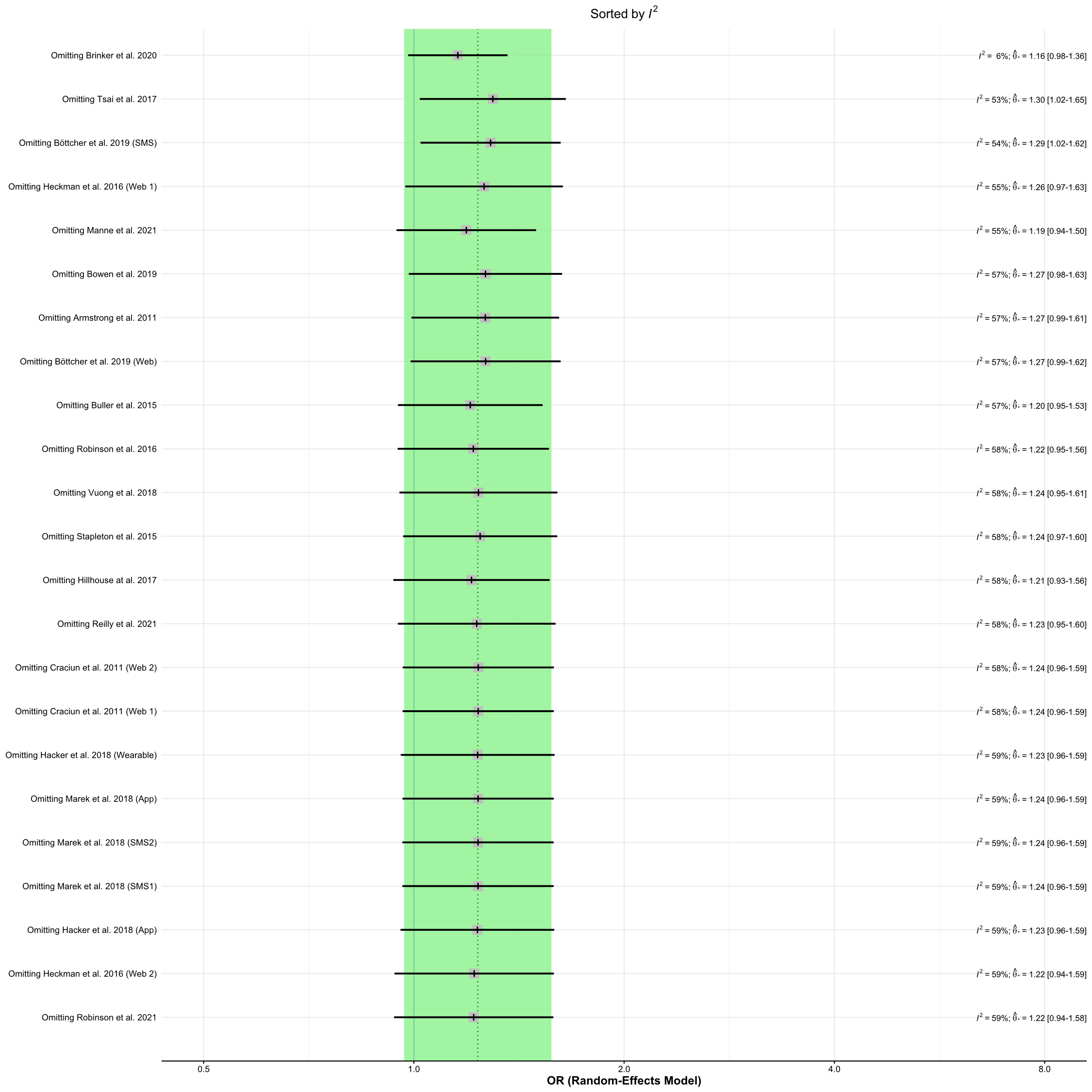


**Figure S6.** Influence graph


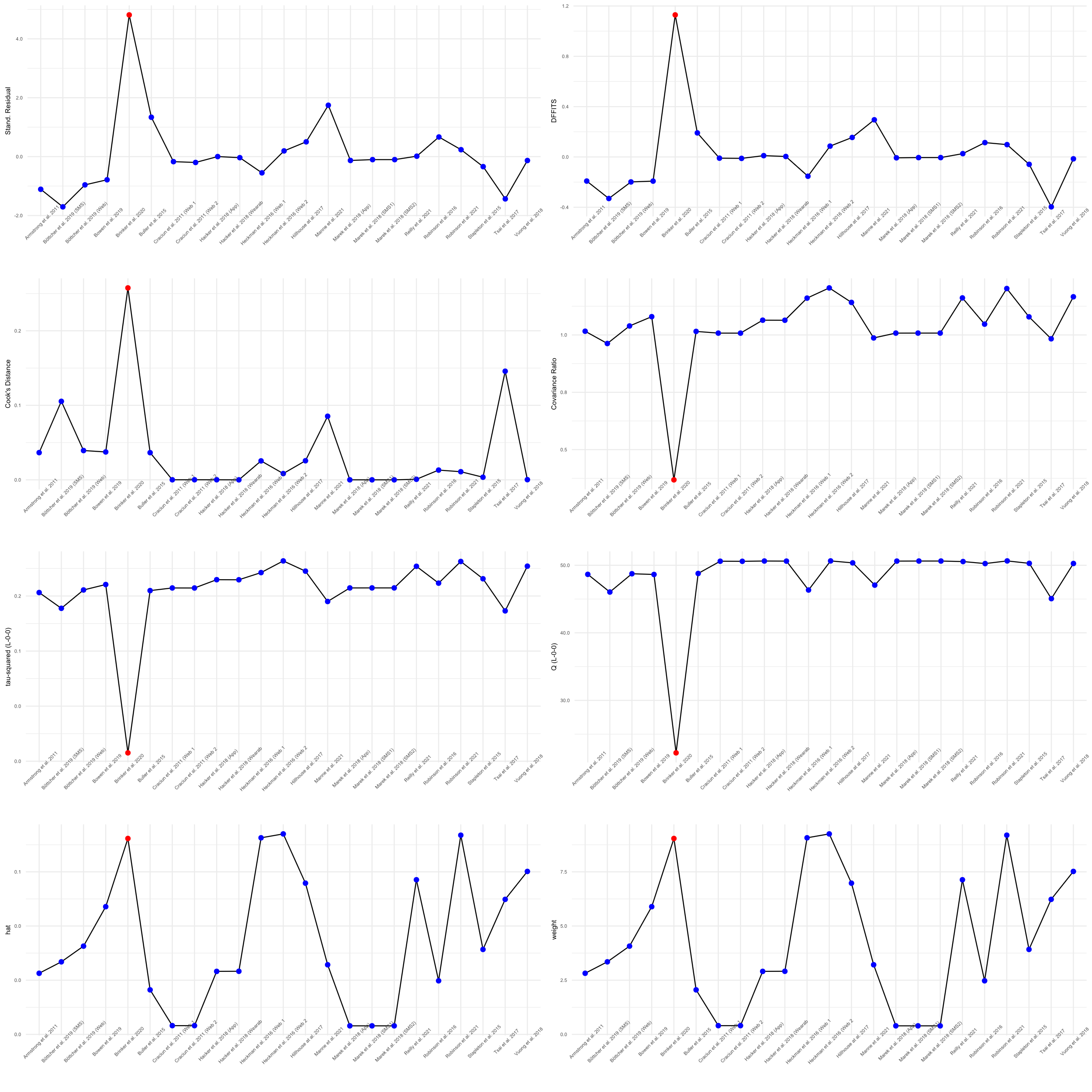


**Figure S7.** Contour enhanced-funnel plot by log odds ratio


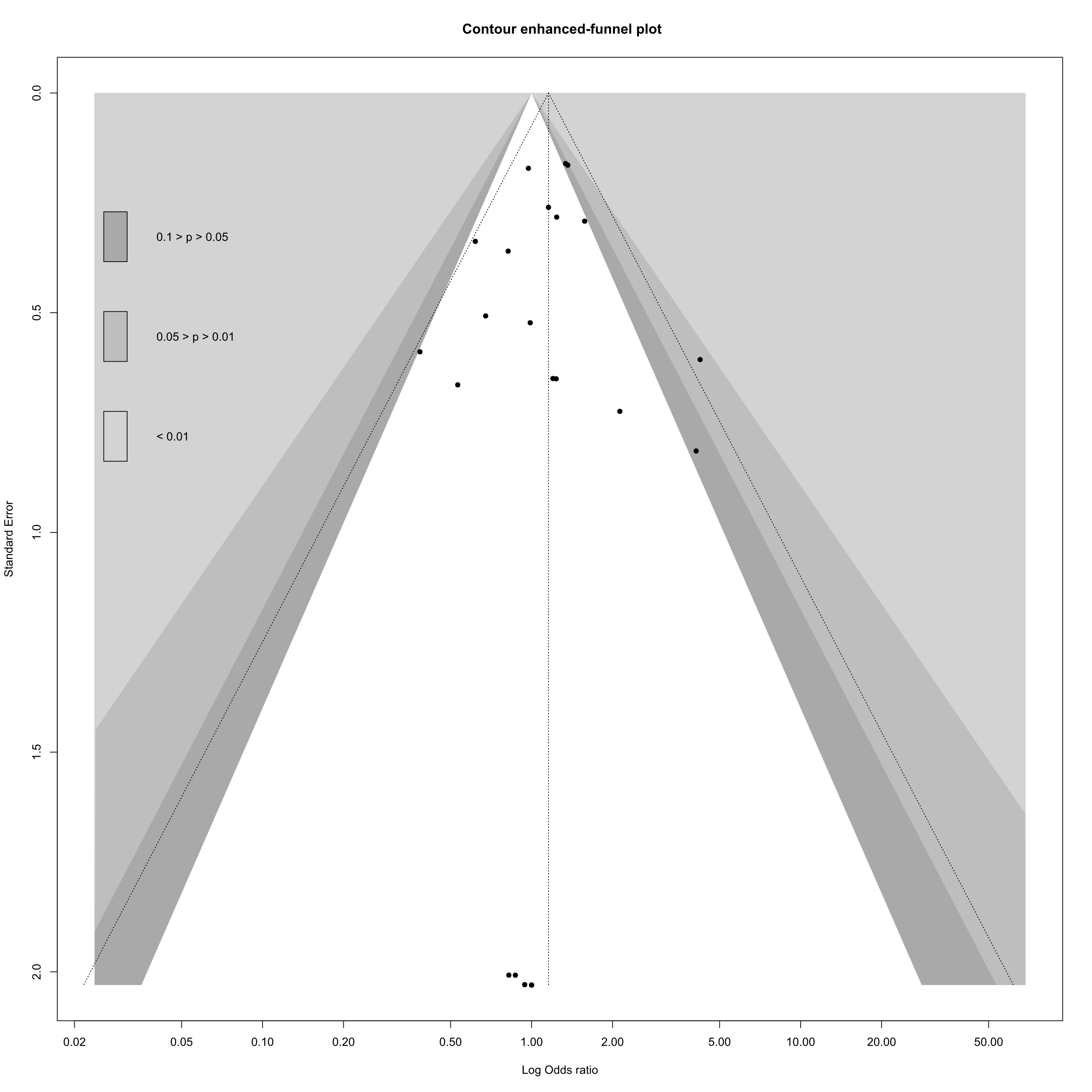


**Figure S8.** Forest plot of proportion subgroup meta-analysis digital health interventions groups


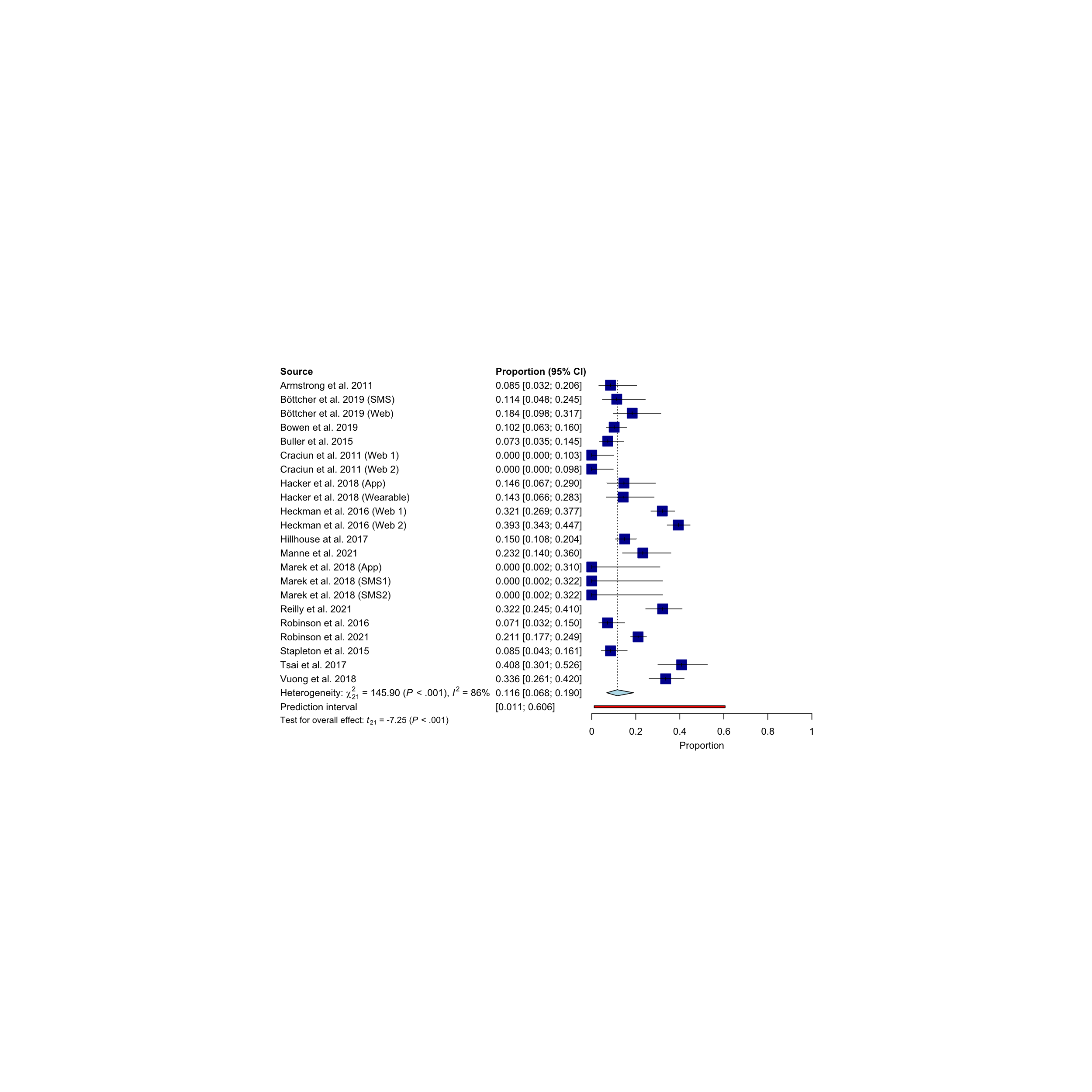


**Figure S9.** Forest plot of proportion subgroup meta-analysis comparator groups


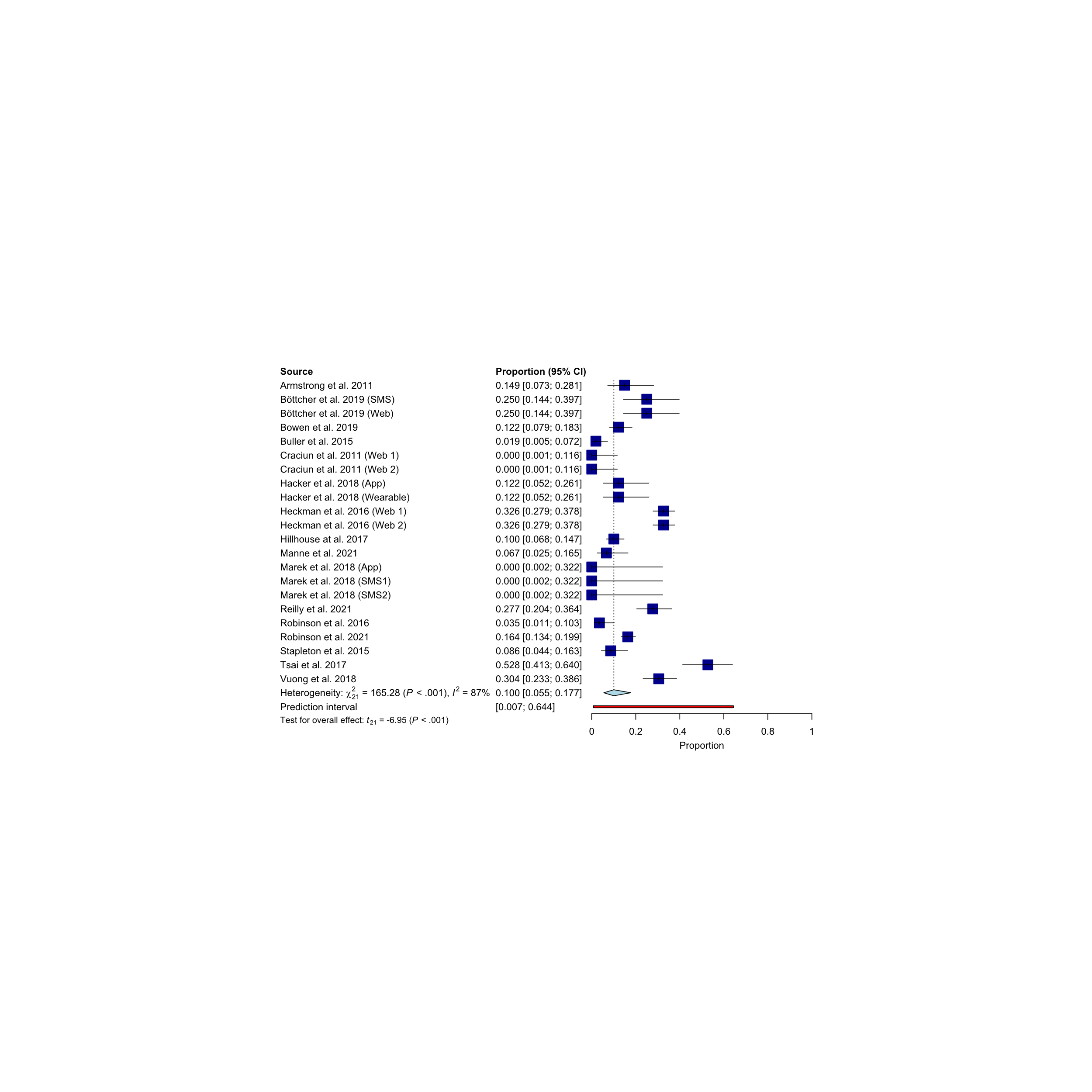


**Figure S10. Forest plot of odds ratio-based subgroup meta-analysis digital health interventions groups**


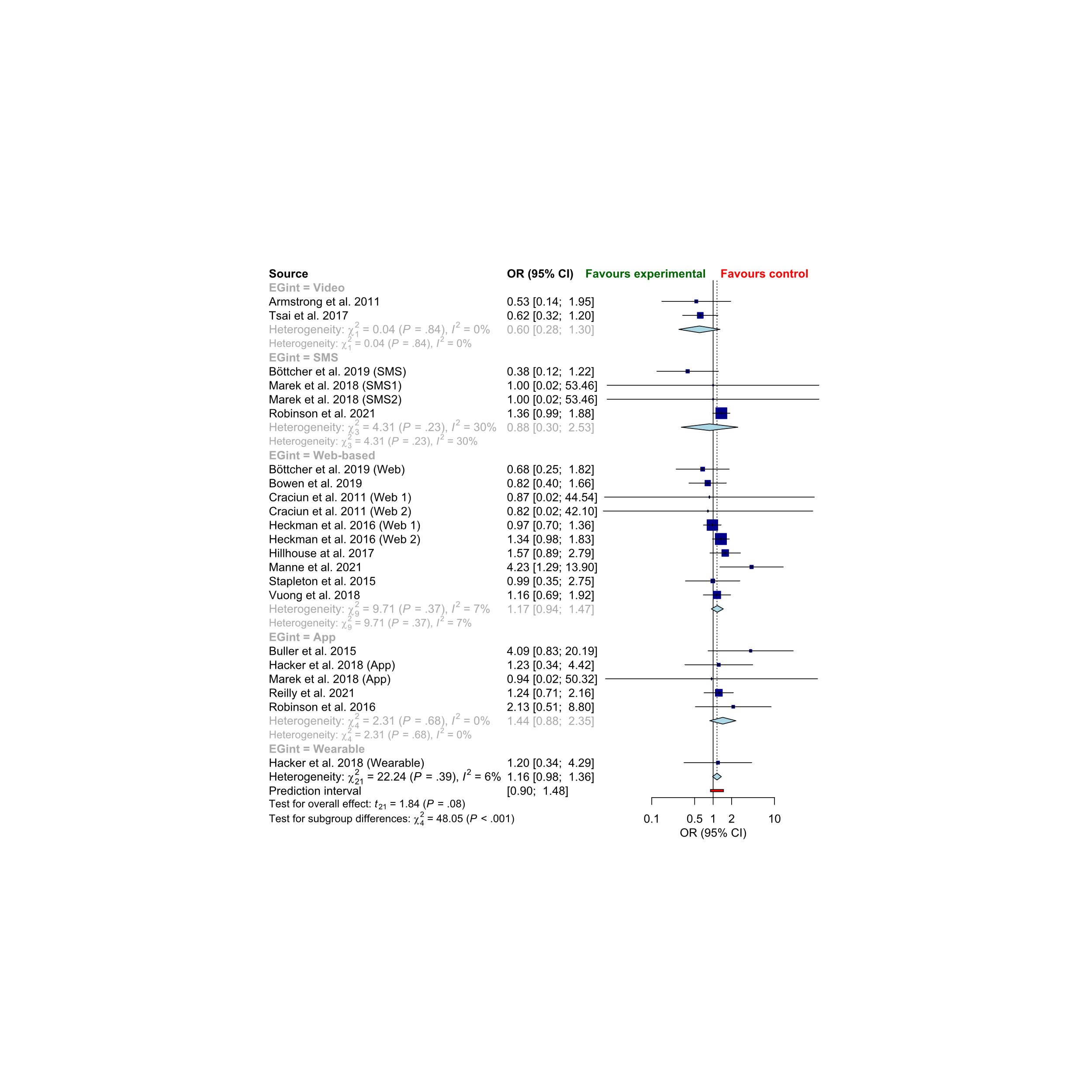

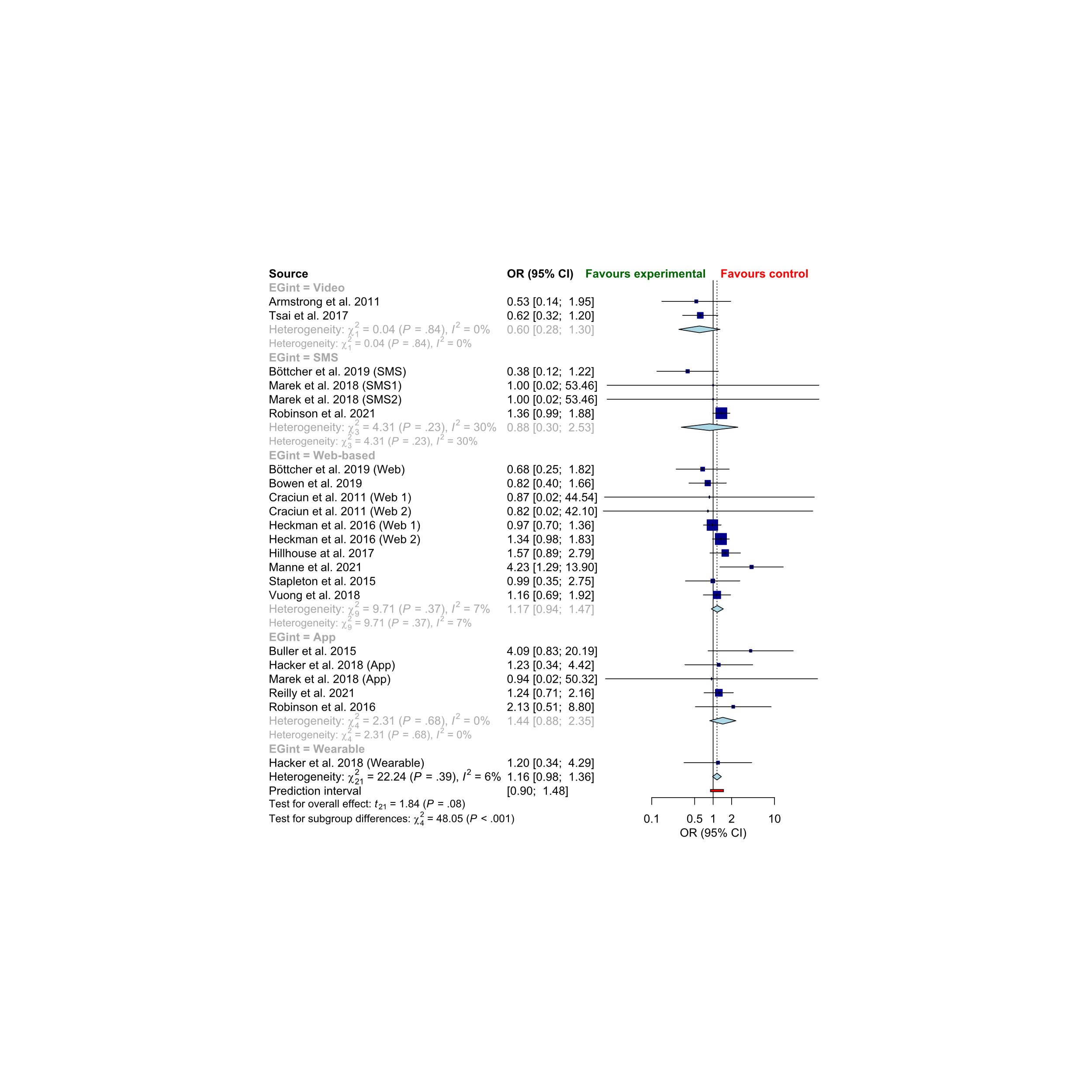

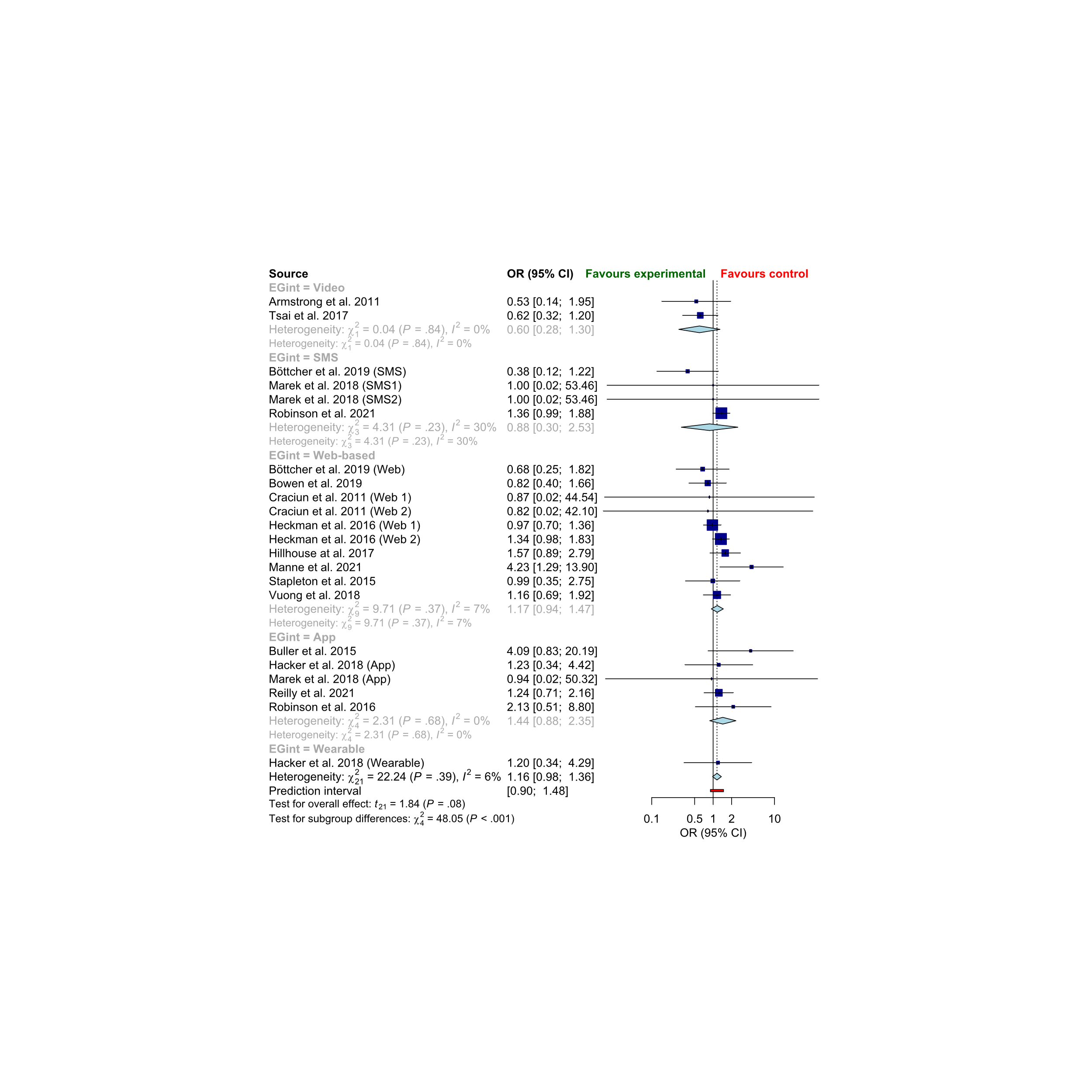


**Figure S11.** Forest plot of odd ratio-based subgroup meta-analysis comparator groups

**
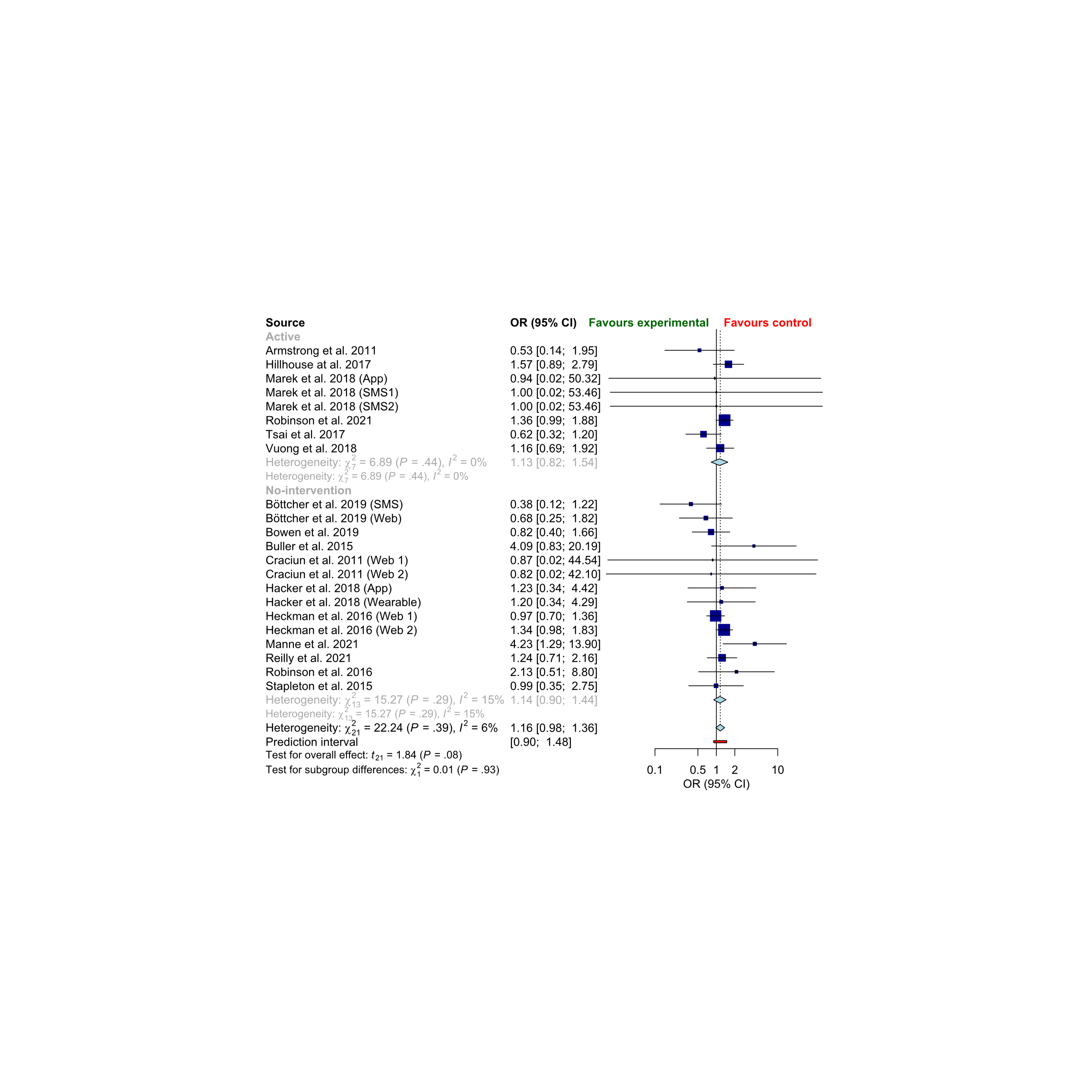
**

**Figure S12.** Bubble plot of association of Participants’ dropout and age


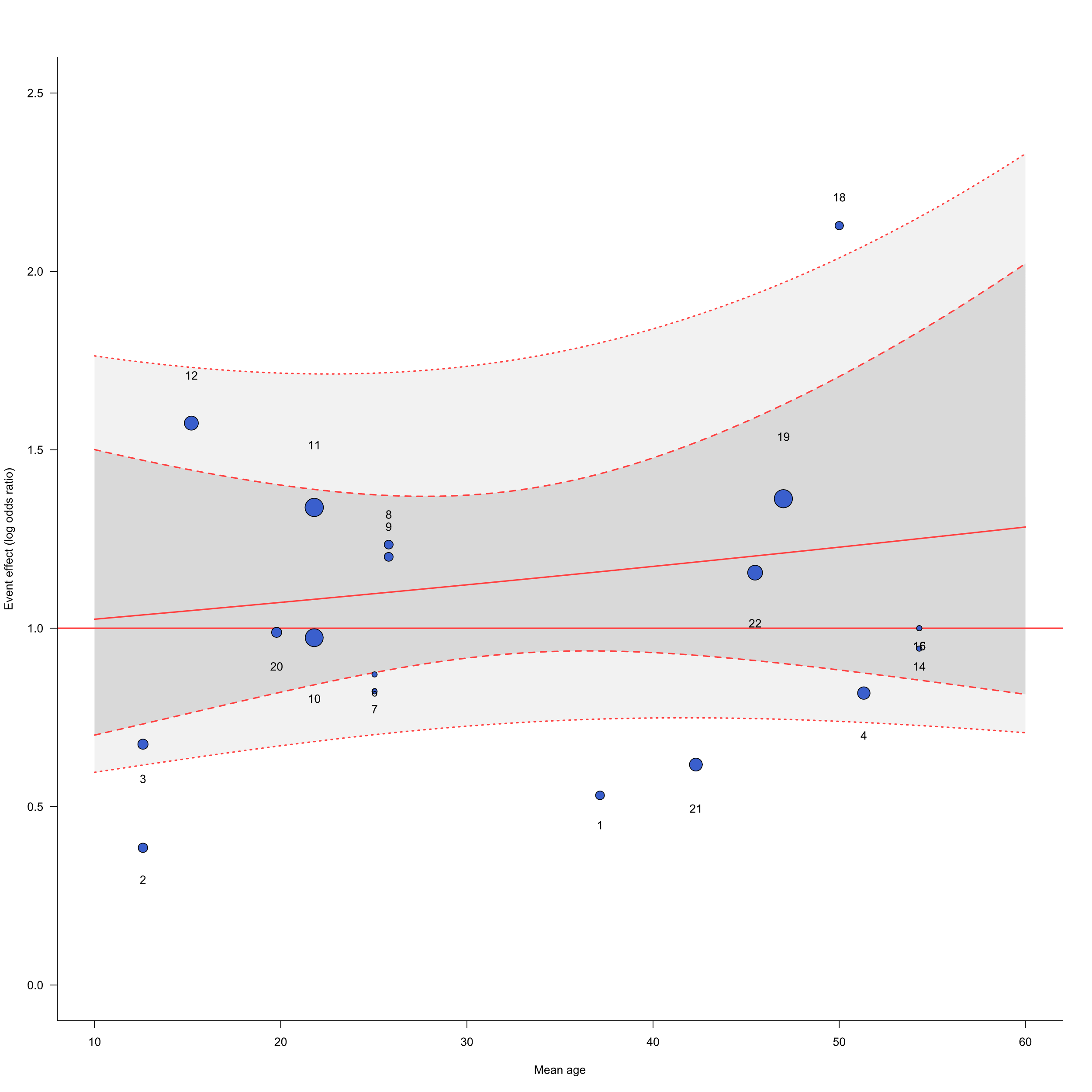

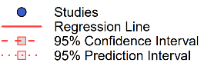


Studies ID: 1. Armstrong et al. 2011; 2. Böttcher et al. 2019 (SMS); 3. Böttcher et al. 2019 (Web); 4. Bowen et al. 2019; 5. Buller et al. 2015; 6. Craciun et al. 2011 (Web 1); 7. Craciun et al. 2011 (Web 2); 8. Hacker et al. 2018 (App); 9. Hacker et al. 2018 (Wearable); 10. Heckman et al. 2016 (Web 1); 11. Heckman et al. 2016 (Web 2); 12. Hillhouse at al. 2017; 13. Manne et al. 2021; 14. Marek et al. 2018 (App); 15. Marek et al. 2018 (SMS1); 16. Marek et al. 2018 (SMS2); 17. Reilly et al. 2021; 18. Robinson et al. 2016; 19. Robinson et al. 2021; 20. Stapleton et al. 2015; 21.Tsai et al. 2017;22. Vuong et al. 2018

**Figure S13.** Bubble plot of association of Participants’ dropout and percentage of females


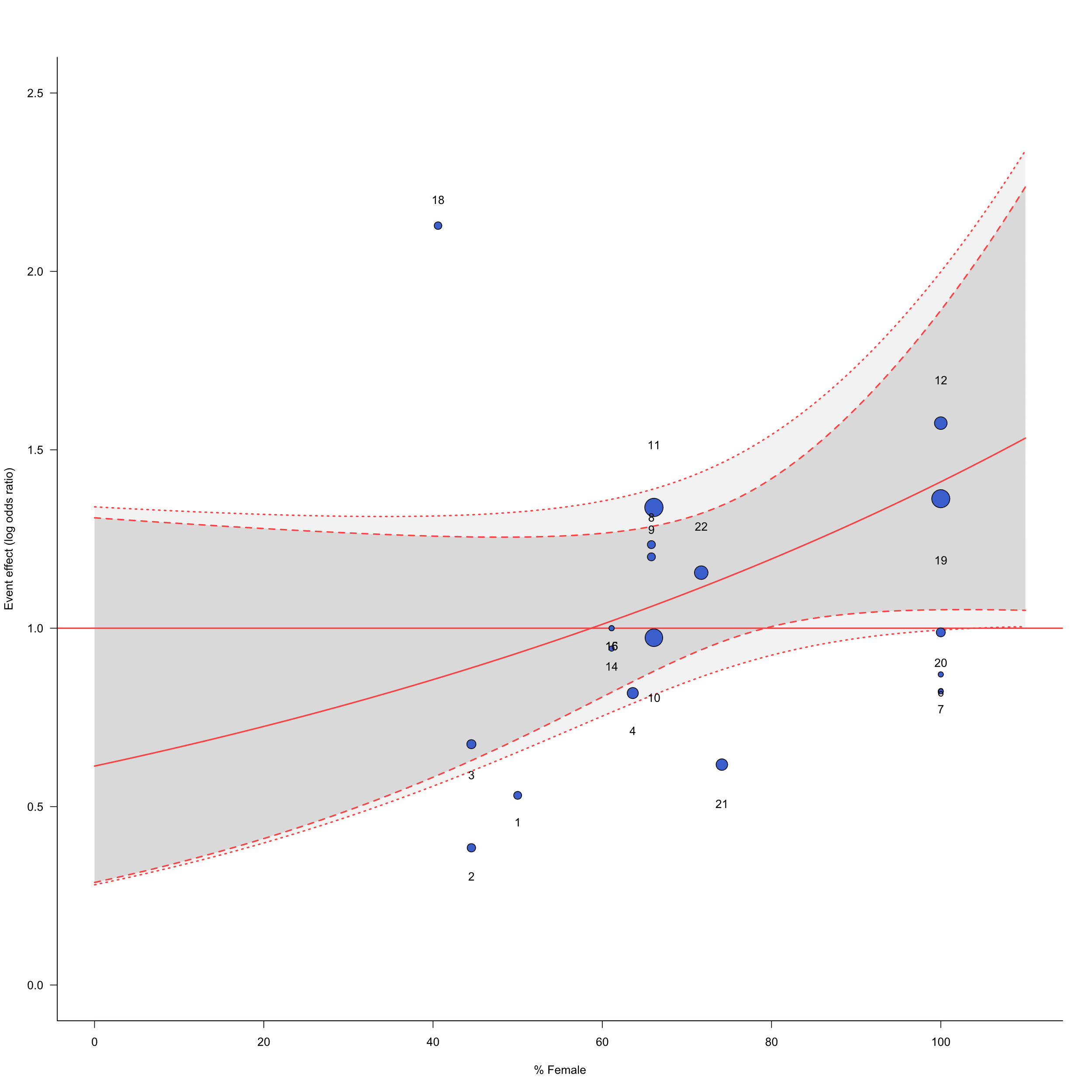

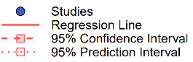


Studies ID: 1. Armstrong et al. 2011; 2. Böttcher et al. 2019 (SMS); 3. Böttcher et al. 2019 (Web); 4. Bowen et al. 2019; 5. Buller et al. 2015; 6. Craciun et al. 2011 (Web 1); 7. Craciun et al. 2011 (Web 2); 8. Hacker et al. 2018 (App); 9. Hacker et al. 2018 (Wearable); 10. Heckman et al. 2016 (Web 1); 11. Heckman et al. 2016 (Web 2); 12. Hillhouse at al. 2017; 13. Manne et al. 2021; 14. Marek et al. 2018 (App); 15. Marek et al. 2018 (SMS1); 16. Marek et al. 2018 (SMS2); 17. Reilly et al. 2021; 18. Robinson et al. 2016; 19. Robinson et al. 2021; 20. Stapleton et al. 2015; 21.Tsai et al. 2017;22. Vuong et al. 2018

**Figure S14.** Bubble plot of association of Participants’ dropout and percentage of males


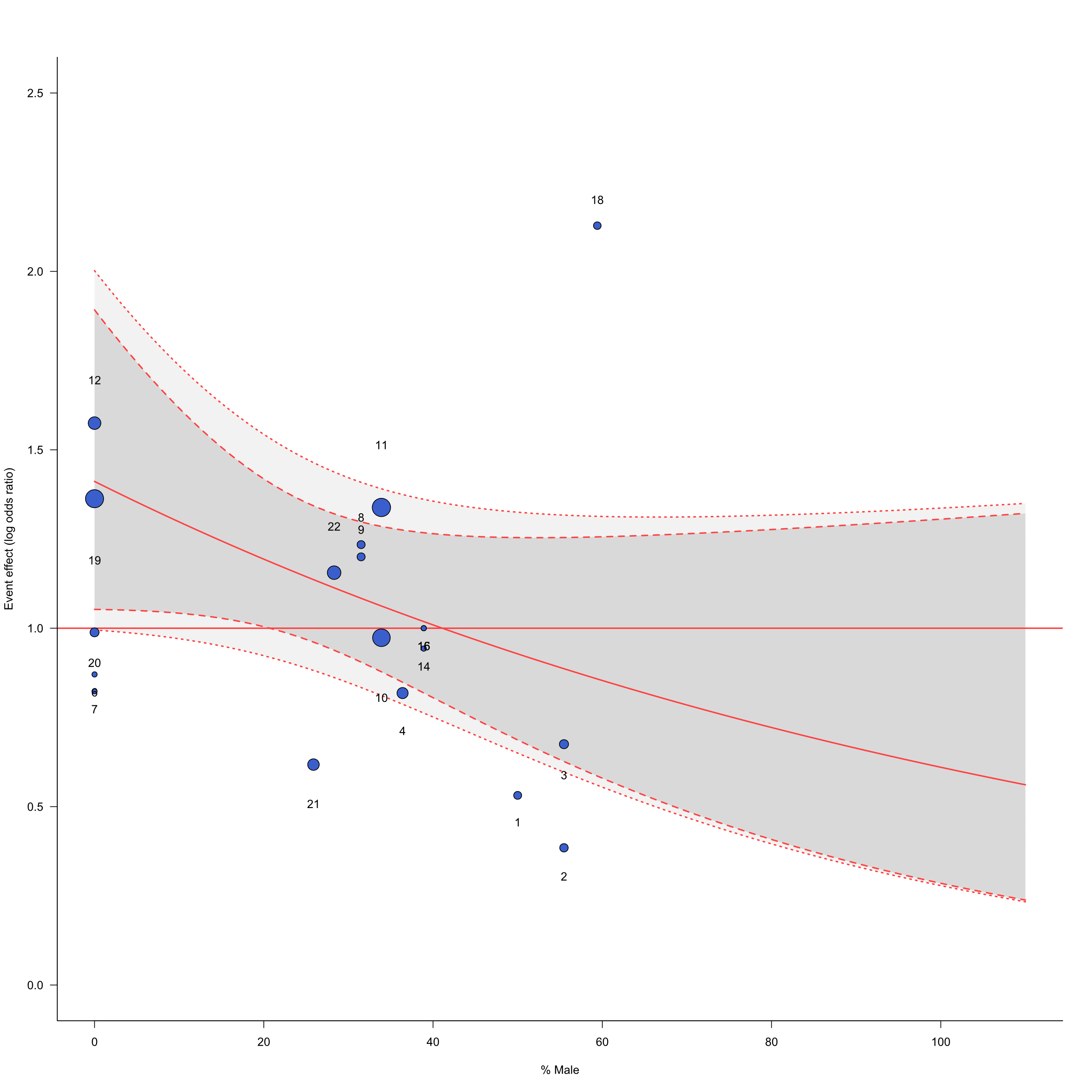

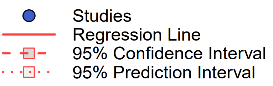


Studies ID: 1. Armstrong et al. 2011; 2. Böttcher et al. 2019 (SMS); 3. Böttcher et al. 2019 (Web); 4. Bowen et al. 2019; 5. Buller et al. 2015; 6. Craciun et al. 2011 (Web 1); 7. Craciun et al. 2011 (Web 2); 8. Hacker et al. 2018 (App); 9. Hacker et al. 2018 (Wearable); 10. Heckman et al. 2016 (Web 1); 11. Heckman et al. 2016 (Web 2); 12. Hillhouse at al. 2017; 13. Manne et al. 2021; 14. Marek et al. 2018 (App); 15. Marek et al. 2018 (SMS1); 16. Marek et al. 2018 (SMS2); 17. Reilly et al. 2021; 18. Robinson et al. 2016; 19. Robinson et al. 2021; 20. Stapleton et al. 2015; 21.Tsai et al. 2017;22. Vuong et al. 2018

**Figure S15.** Bubble plot of association of Participants’ dropout and length of intervention (months)


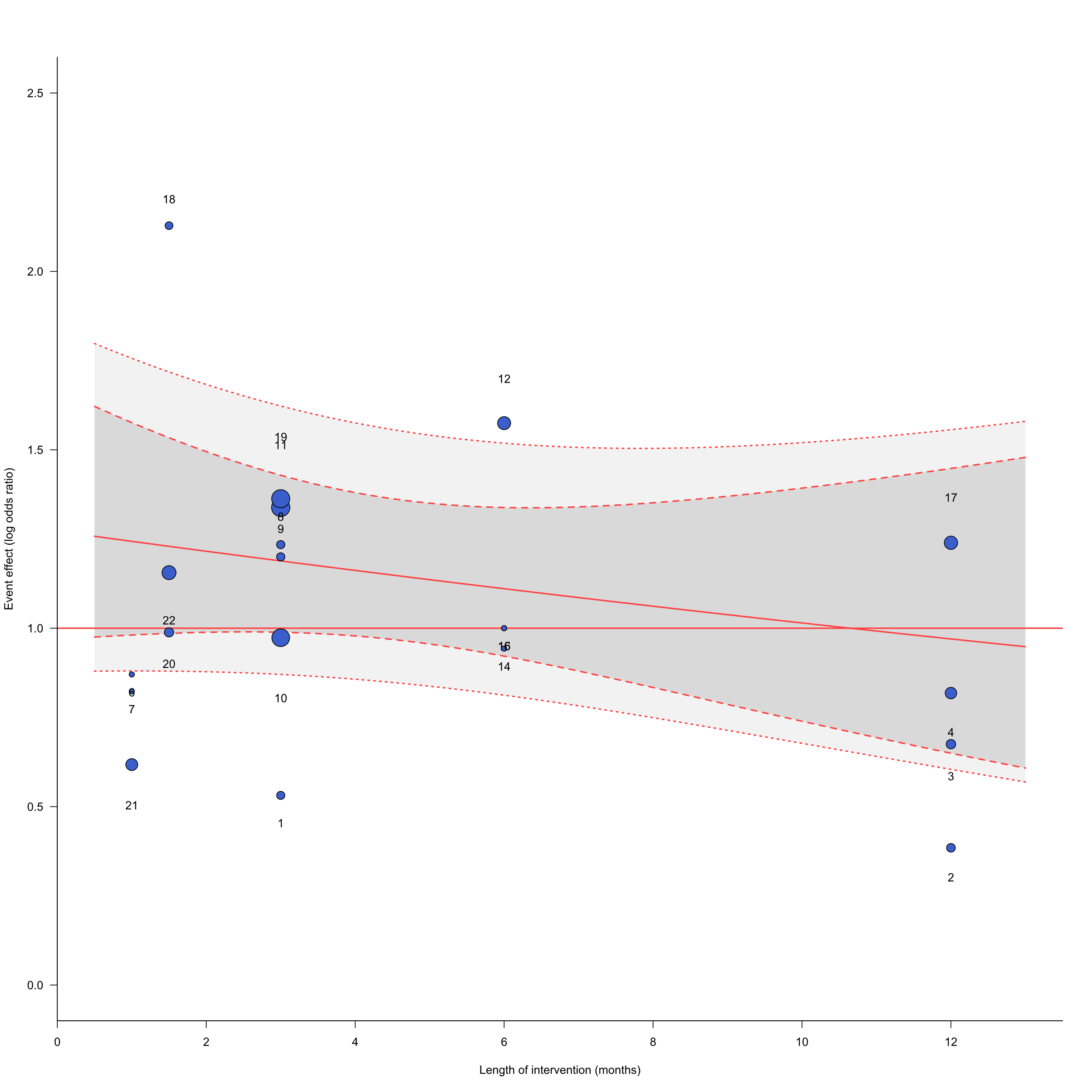

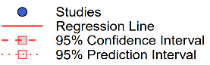


Studies ID: 1. Armstrong et al. 2011; 2. Böttcher et al. 2019 (SMS); 3. Böttcher et al. 2019 (Web); 4. Bowen et al. 2019; 5. Buller et al. 2015; 6. Craciun et al. 2011 (Web 1); 7. Craciun et al. 2011 (Web 2); 8. Hacker et al. 2018 (App); 9. Hacker et al. 2018 (Wearable); 10. Heckman et al. 2016 (Web 1); 11. Heckman et al. 2016 (Web 2); 12. Hillhouse at al. 2017; 13. Manne et al. 2021; 14. Marek et al. 2018 (App); 15. Marek et al. 2018 (SMS1); 16. Marek et al. 2018 (SMS2); 17. Reilly et al. 2021; 18. Robinson et al. 2016; 19. Robinson et al. 2021; 20. Stapleton et al. 2015; 21.Tsai et al. 2017;22. Vuong et al. 2018

**Figure S16.** Bubble plot of association of Participants’ dropout and sample size


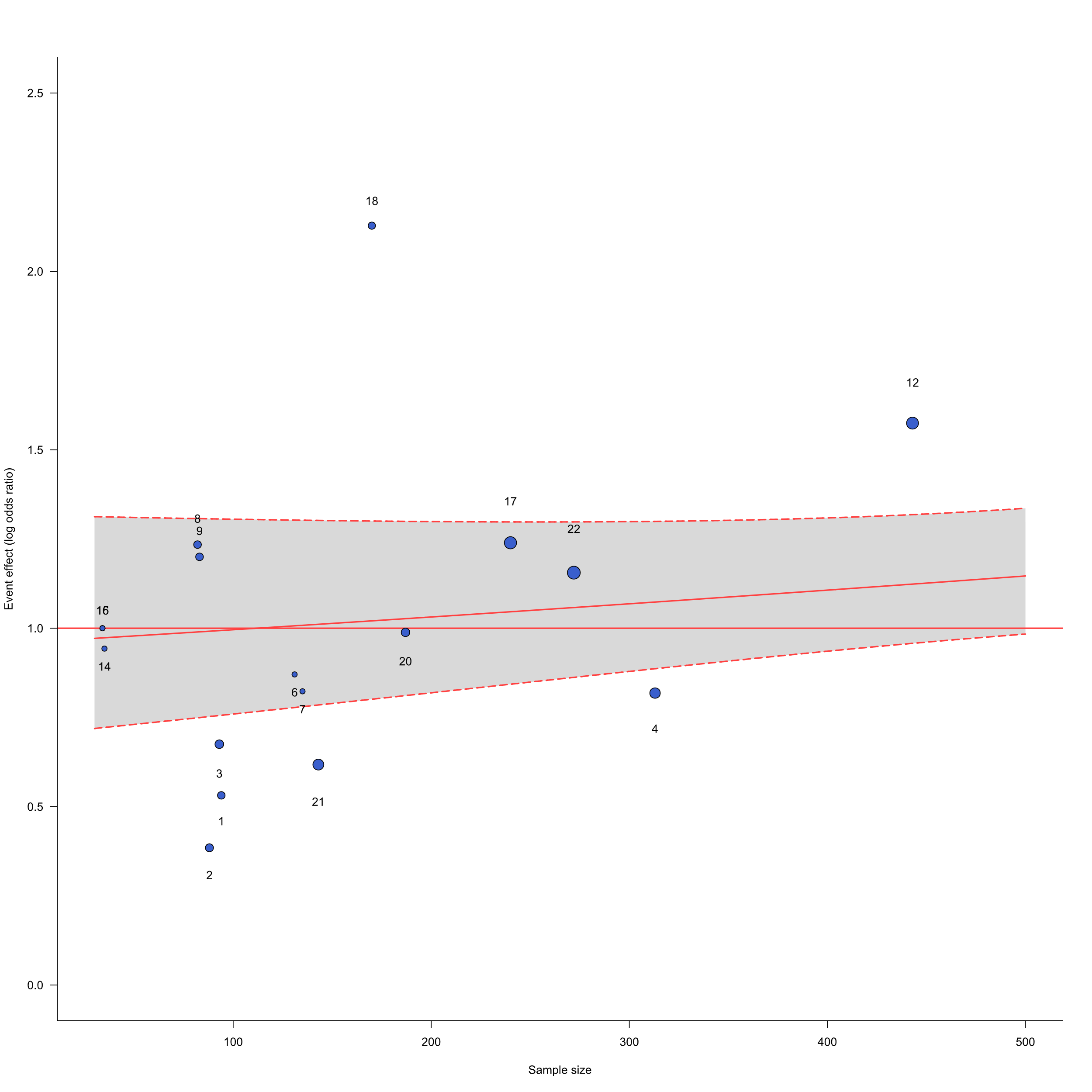

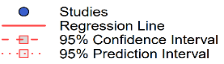


Studies ID: 1. Armstrong et al. 2011; 2. Böttcher et al. 2019 (SMS); 3. Böttcher et al. 2019 (Web); 4. Bowen et al. 2019; 5. Buller et al. 2015; 6. Craciun et al. 2011 (Web 1); 7. Craciun et al. 2011 (Web 2); 8. Hacker et al. 2018 (App); 9. Hacker et al. 2018 (Wearable); 10. Heckman et al. 2016 (Web 1); 11. Heckman et al. 2016 (Web 2); 12. Hillhouse at al. 2017; 13. Manne et al. 2021; 14. Marek et al. 2018 (App); 15. Marek et al. 2018 (SMS1); 16. Marek et al. 2018 (SMS2); 17. Reilly et al. 2021; 18. Robinson et al. 2016; 19. Robinson et al. 2021; 20. Stapleton et al. 2015; 21.Tsai et al. 2017;22. Vuong et al. 2018
